# Rational design of triazole tyrosinase inhibitors via integrated free energy calculations

**DOI:** 10.1007/s11030-026-11601-7

**Published:** 2026-05-29

**Authors:** Nathália Luiza Oliveira Costa, Cláudio Nahum Alves, Lucas Sousa Martins, José Rogério A. Silva

**Affiliations:** 1 Laboratory of Computer Modeling of Molecular Biosystems (CompMBio), Federal University of Pará, Belém, Pará 66075-110 Brazil; 2 Laboratório de Planejamento e Desenvolvimento de Fármacos, Instituto de Ciências Exatas e Naturais, Universidade Federal do Pará, Belém, Pará 66075- 110 Brazil; 3 Catalysis and Peptide Research Unit, University of KwaZulu-Natal, Durban, 4000 South Africa

**Keywords:** Tyrosinase Inhibition, Free energy perturbation, Linear interaction energy, Molecular dynamics, Drug design, Hyperpigmentation.

## Abstract

Tyrosinase (TYR) is a copper-containing metalloenzyme involved in melanin biosynthesis and is an important target in the development of treatments for hyperpigmentation disorders, including melasma, post-inflammatory hyperpigmentation, and melanoma. Among the scaffold classes investigated for TYR inhibition, 1,2,4-triazole derivatives have attracted increasing interest. In this study, we evaluated a structurally diverse series of triazole derivatives using an integrated computational workflow that included absorption, distribution, metabolism, excretion and toxicity (ADMET) profiling, molecular docking, molecular dynamics (MD) simulations, and binding free energy calculations based on Linear Interaction Energy (LIE) and Free Energy Perturbation (FEP). Using *Bacillus megaterium* TYR as the model enzyme, the triazole derivatives generally produced more favorable docking scores than kojic acid and established recurring interactions with hydrophobic residues such as Met215, Val217, Ala221, and Phe227, as well as hydrogen-bonding contacts with Gly216. The free energy calculations reproduced the experimental binding affinities with high accuracy (FEP: R² = 0.91, MAE = 0.20 kcal/mol; LIE: R² = 0.93, MAE = 0.54 kcal/mol). Residue-level energy analysis indicated that interactions involving Arg209, Gly216 and Val218 play a major role in ligand binding across the series, with aromatic and halogenated substituents favoring stronger van der Waals contacts in this region. Overall, these results provide a consistent structural and energetic framework for understanding triazole-based TYR inhibition and may support the rational design of improved inhibitors for cosmetic and therapeutic applications.

**Supplementary Information:**

The online version contains supplementary material available at https://doi.org/10.1007/s11030-026-11601-7.

## Introduction

**Fig. 1:**
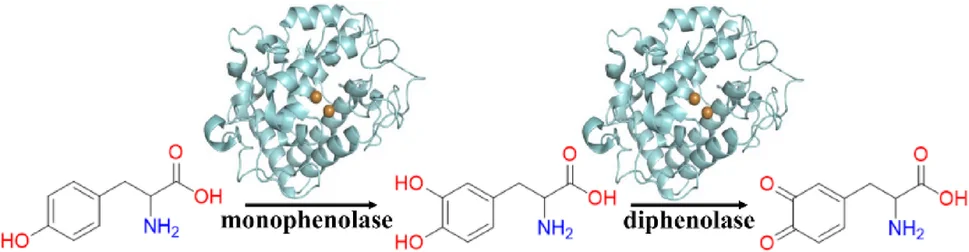
Schematic overview of the reactions catalyzed by TYR during melanogenesis

Tyrosinase (TYR, EC 1.14.18.1) is a binuclear copper metalloenzyme that plays a central role in melanogenesis by catalyzing the hydroxylation of L-tyrosine to L-DOPA and the subsequent oxidation of L-DOPA to dopaquinone, a key intermediate in melanin biosynthesis [1–6] (Fig. 1). Because excessive melanin production is associated with dermatological conditions such as melasma, post-inflammatory hyperpigmentation, lentigo, freckles, nevi, and chloasma gravidarum, TYR inhibition has become an important strategy for the development of cosmetic and therapeutic depigmenting agents [7–9].

Beyond its dermatological relevance, TYR also provides a useful biochemical model for studying type-III copper enzymes and ligand recognition in metalloenzyme active sites [1, 10]. Therefore, the identification of structurally diverse TYR inhibitors remains relevant not only for the development of skin-lightening agents but also for understanding how small molecules engage the conserved binuclear copper catalytic pocket.

Currently, only a limited number of agents are prescribed or available over the counter for the treatment of skin hyperpigmentation [11]. These include kojic acid (KA), hydroquinone, arbutin, tropolone and azelaic acid. However, all of these compounds have limitations in terms of efficacy and safety [12]. KA is among the most widely used ingredients in cosmetic formulations [13]. However, like other depigmenting agents, it is associated with cytotoxicity and chemical instability [13]. KA in particular is limited by its low molecular weight, high toxicity, unfavorable lipophilicity, and poor formulation stability. Together, these features compromise its ADME profile, may trigger adverse effects, and restrict its broader application in cosmetic products [14].

These limitations highlight the need for structurally distinct TYR inhibitors with improved pharmacological profiles [15]. Recent experimental studies have shown that novel hybrid scaffolds incorporating established pharmacophoric elements can display inhibitory potency 10–30-fold greater than that of KA [16], reinforcing the rationale for exploring structurally diverse TYR inhibitor chemotypes. Heterocyclic compounds represent an important class of molecules in medicinal chemistry because of their broad structural diversity and frequent occurrence in bioactive compounds. Today, most accessible drugs consist of heterocyclic systems [17]. Many N-heterocyclic compounds found in nature exhibit relevant physiological and pharmacological properties [18]. Triazole derivatives (TRDs) have emerged as promising TYR inhibitors with potential relevance to dermatological disorders, including post-inflammatory hyperpigmentation, melasma, and melanoma [19, 20]. Azoles are five-membered nitrogen-containing heterocycles with electron-rich properties that have attracted considerable attention in pharmaceutical and medicinal chemistry research [21]. Within this class, several subgroups exhibit potent inhibitory activity, including imidazoles, pyrazoles, triazoles, tetrazoles, oxadiazoles, thiadiazoles and thiazoles.

In the present study, we focus on the computational analysis of triazole derivatives, an important subgroup within the broader class of five-membered N-heterocyclic compounds. These derivatives have been extensively investigated as TYR inhibitors and several studies have reported relevant findings regarding their inhibitory activity and structure–activity relationships [22–24]. In particular, previous reports have described three distinct sets of 1,2,4-triazole derivatives (hereafter referred to as “TzDs”), each containing different substituent patterns at multiple positions in the molecular scaffold. Among these, triazole derivatives have emerged as promising lead structures for the development of depigmenting agents targeting hyperpigmentation and other dyschromias [22, 25–27].

Although several computational studies have investigated TYR inhibitors, most have relied primarily on docking, QSAR, or endpoint rescoring approaches, which provide useful but limited information on binding affinity because they only partially account for protein flexibility, solvent reorganization, and ligand-specific energetic effects [28]. In this context, the combined use of Free Energy Perturbation (FEP) and Linear Interaction Energy (LIE) offers two complementary levels of analysis. FEP was used here for a structurally controlled subset of congeneric TzDs, allowing quantitative evaluation of relative binding free-energy changes associated with specific halogen substitutions. In contrast, LIE was applied to a broader subset of ligands to estimate absolute binding free energies and to provide an interpretable energetic framework for residue-level analysis. Thus, FEP and LIE were not used as redundant methods. However, as complementary tools, FEP provides rigorous relative free-energy validation within a chemically consistent perturbation network, whereas LIE expands the analysis across a broader chemical space and supports mechanistic interpretation of binding determinants. To our knowledge, this is the first study to integrate these two free-energy approaches to rationalize the potency of a diverse TzD dataset against TYR.

In this study, we employed molecular modeling approaches to support the design of TYR inhibitors, including molecular docking, molecular dynamics simulations and free energy calculations. These methods were used to investigate the interactions of TzDs with TYR from *Bacillus megaterium*, and to characterize the inhibition mechanism through structural and energetic analyses consistent with the available experimental data. All computational procedures adopted in this study were based on previously validated protocols reported in the literature [29–33].

## Materials and methods

### ADMET analysis and molecular docking

Before performing molecular docking, ADMET (absorption, distribution, metabolism, excretion, and toxicity) profiles of TzDs were predicted using in silico methods. Pharmacokinetic parameters were predicted with SwissADME [34] to assess absorption, distribution, metabolism, and excretion (ADME) properties relevant to drug-like behavior. Integrating ADMET profiling with molecular docking enables prioritizing compounds with favorable pharmacokinetic profiles and strong predicted binding, thereby supporting the rational drug design process. The Supporting Information file provides the SMILES codes for the selected 48 TzDs (Table S1).

The 3D structure of the TYR enzyme from *Bacillus megaterium* (TYR*Bm*) complexed with the crystallographic inhibitor kojic acid (KA) was obtained from PDB entry 5I38 [35], with a resolution of 2.60 Å. This structure was selected over *Agaricus bisporus* TYR (TYR*Ab*, PDB ID: 2Y9X) [36] because of its higher resolution and more complete structural representation of the catalytic pocket. The TYR*Bm* structure preserves the canonical His–Cu coordination environment and exhibits high active-site similarity with TYR*Ab* as indicated by sequence alignment and structural superposition [37]. The Molegro Virtual Docker (MVD) program (version 5.5) and the MOLDOCK scoring function [38], which have been successfully applied in previous TYR investigations [29–31], were used to dock the TzDs (Table 1) into the TYR active site. In MVD, a sphere with a radius of 12 Å and a resolution of 0.20 Å was centered on the KA coordinates, specifically at 1.52, 101.99, and 25.06 Å. At this stage, the copper ions were modeled as van der Waals (vdW) spheres at the active site of TYR using the Lennard-Jones (LJ) 12 − 6 potential [39]. This docking protocol was previously validated by redocking KA into TYR*Bm* (PDB ID 5I38), as detailed in Martins et al. [29–31], confirming that the MOLDOCK scoring function reliably reproduces the experimentally observed binding mode of KA within an accepted RMSD threshold. The same validated setup was therefore applied without modification to the docking of the TzD series.

A total of 48 TzDs with reported inhibitory activity against TYR were selected from the literature for ADME profiling and molecular docking (Table 1) [22–24, 26]. These 48 compounds represent the complete set of 1,2,4-triazole derivatives for which both experimental IC₅₀ values against tyrosinase and a fully defined chemical structure were available at the time of study design, as identified through a systematic search of Ghani’s comprehensive review [25], cross-referenced with the primary experimental literature [22–24, 26]. Compounds for which IC_50_ values were reported only as ranges, or without a precise experimental value, were excluded from the free-energy calculations, as such data cannot be converted reliably to ΔG values. According to this classification, the compounds were organized into five distinct groups, allowing for focused structural and activity-based comparisons [25]: (1) TzDs bearing substituents at the R_2_ position, such as (A) methoxyphenyl or (B) butyl groups [26]; (2) TzD–semicarbazide hybrids, featuring semicarbazide moieties [22]; (3) TzD–thiosemicarbazide hybrids, containing thiosemicarbazide fragments [22]; (4) Aryl phenoxymethyl–TzDs, conjugated with thiosemicarbazide groups [23] and (5) Bi-heterocyclic acetamide derivatives, incorporating both TzD and aminothiazole rings [24].

**Table 1:**
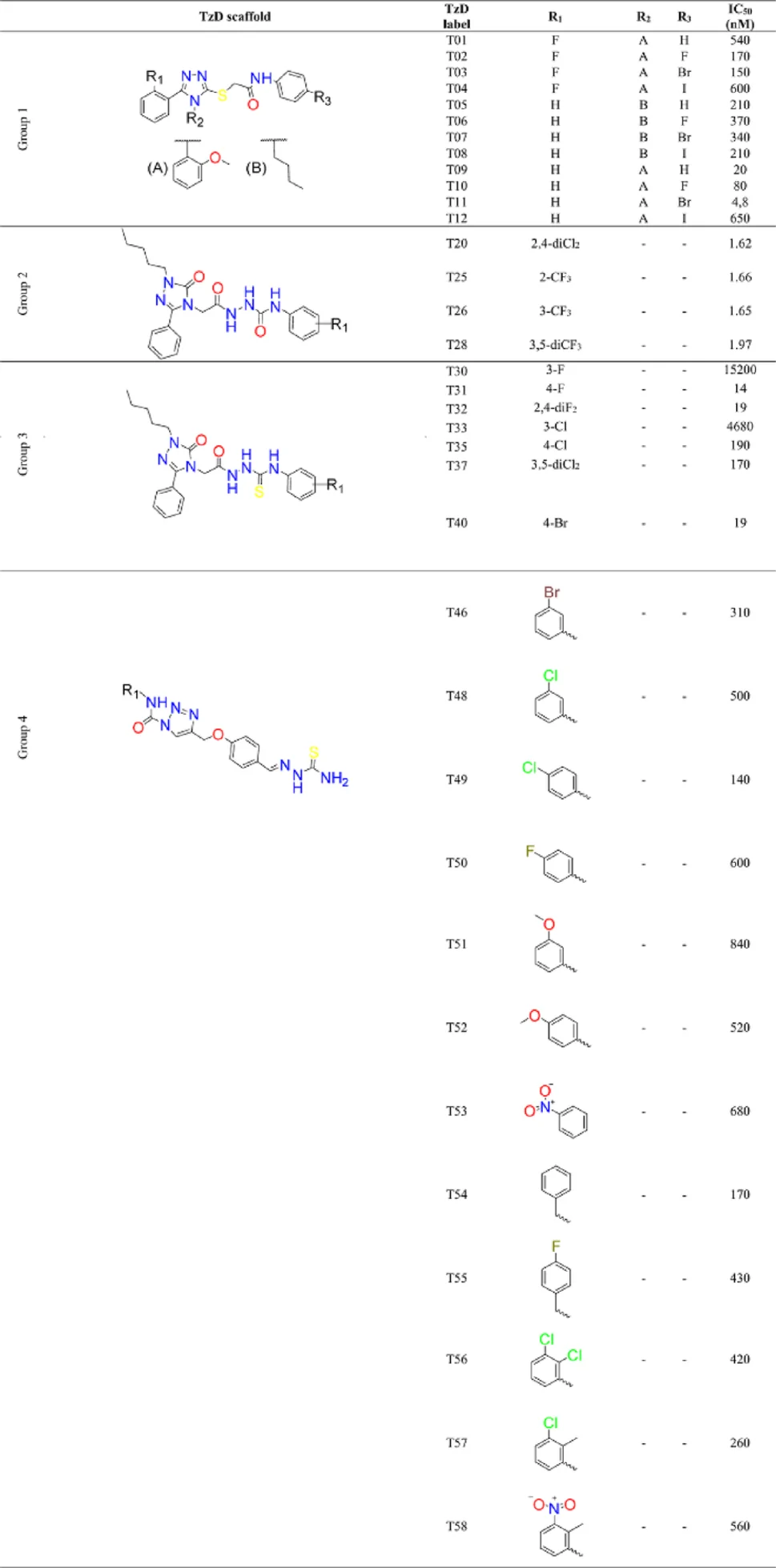

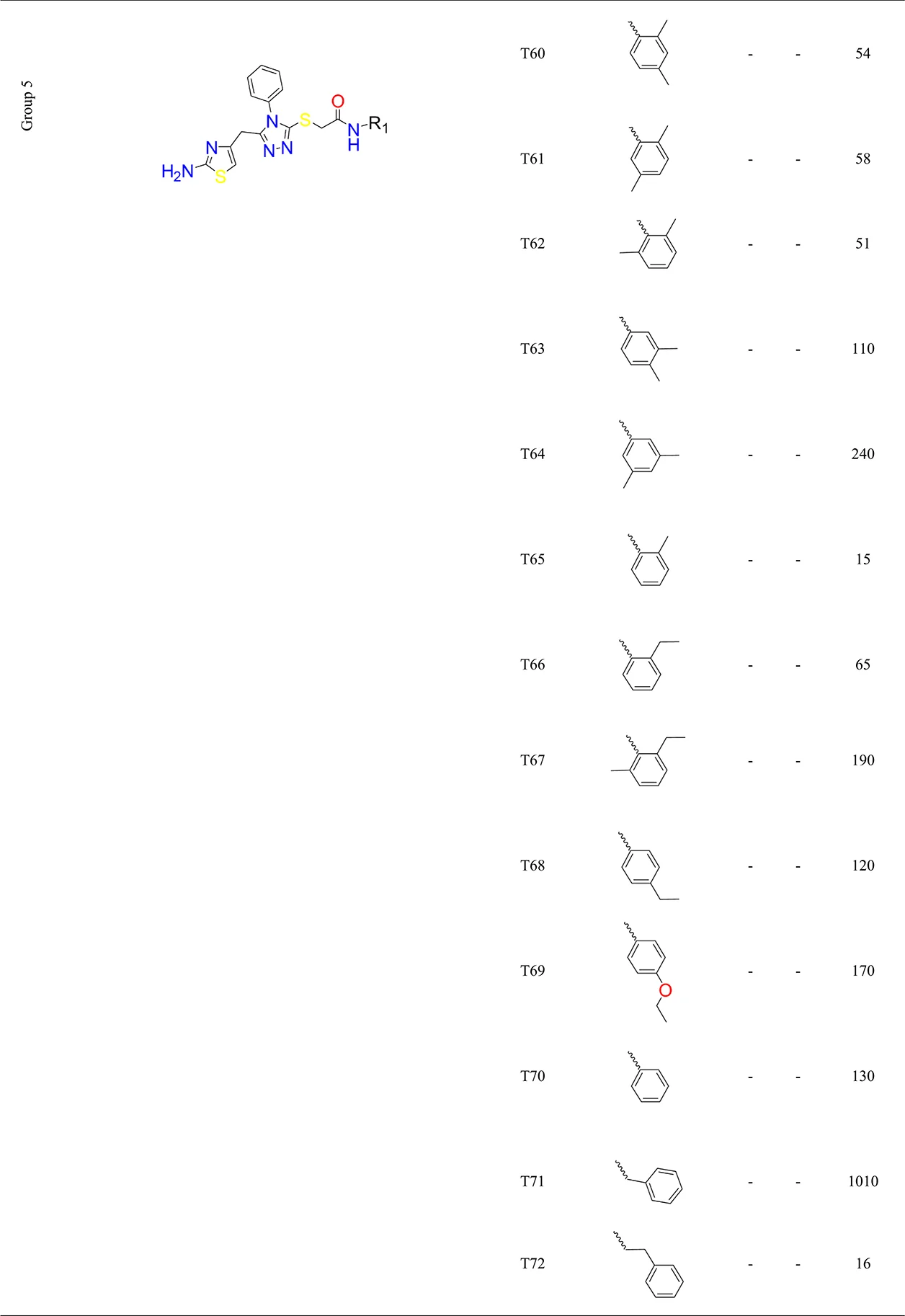
2D chemical structures of the 48 TzDs evaluated in this study, along with their experimentally reported IC₅₀ values (in nM) for TYR inhibition

## MD and free energy calculations

### System setup for LIE

The FEP and LIE calculations were designed to answer different but complementary questions. FEP was used to quantify relative binding free-energy changes within a small, congeneric subset of TzDs, enabling chemically meaningful alchemical transformations with minimal perturbation of the common scaffold. This approach is particularly suitable for testing whether the docking-derived binding mode can reproduce subtle affinity shifts caused by halogen substitutions [40]. LIE, in turn, was used to extend the energetic analysis to a larger and more structurally diverse set of TzDs. Unlike FEP, LIE provides absolute binding free-energy estimates and separates the van der Waals and electrostatic components of ligand binding, enabling residue-level interpretation of the main interactions that govern activity trends [41]. Therefore, the two methods were used in a complementary manner: FEP for rigorous relative validation within a controlled perturbation network, and LIE for broader ranking and mechanistic interpretation.

Following molecular docking, 15 TYR-TzD systems were prepared for MD simulations using the Q6 software [42]. Initially, ionizable residues located outside the spherical boundary or near the edge of the simulation region were modeled in their neutral forms. In contrast, ionizable residues within the simulation sphere were protonated based on pKa predictions from the PDB2PQR server [43] using the PROPKA algorithm [44].

The OPLS-AA force field, which includes improved protein torsional parameters [45], was adopted for the receptor. The metal center in the active site of TYR was treated using the Cu²⁺ dummy model proposed by Liao et al. [46] TzD parameters compatible with this force field were obtained using Schrödinger’s MACROMODEL package (version 2019-4) [47] and translated into the Q format. The TIP3P water model [48] was selected to represent the solvent environment, and solvent bond lengths and angles were constrained using the SHAKE algorithm [49].

All systems were solvated with a 20 Å radius simulation sphere of TIP3P water molecules, centered on the ligand’s center of mass. To ensure stability throughout the simulations, the sphere’s geometric center was restrained to a central atom of the ligand. The spherical boundary was treated using the surface-constrained all-atom solvent (SCAAS) model [50], which applies polarization and radial constraints at the sphere’s surface to represent bulk water behavior accurately.

Subsequently, each system underwent the following MD simulation phases: (1) heating, (2) equilibration, and (3) production. Nonbonded interaction energies were computed using a 10 Å cutoff, with ligand atoms excluded from this restriction. Long-range electrostatic interactions were calculated through the local reaction field (LRF) multiple expansion approach [51]. The nonbonded pair lists and interaction energy values between the ligand and its surroundings were saved every 25 steps. All atoms outside the 20 Å radius simulation sphere were restrained to their initial positions.

The MD protocol began with 5000 steps using a very short time step (0.1 fs) and a temperature of 1 K, coupled to a strong thermal bath (0.1 fs coupling). In this initial stage, heavy atoms were restrained using a force constant of 50 kcal/(mol·Å²). Subsequently, each system was gradually heated to 310 K over 100 ps, using a time step of 1 fs and a bath coupling of 100 fs. The restraints applied to the solute heavy atoms were gradually released.

For each system, 10 independent 2 ns MD replicas were run at 310 K for production sampling, using different initial velocities generated from the Maxwell–Boltzmann distribution. The time step was set to 2 fs, and all bonds involving hydrogen atoms were constrained using the SHAKE algorithm [49]. This simulation length should be interpreted in the context of the Q6/SCAAS protocol rather than as a conventional full-protein periodic-boundary MD simulation [52–54]. In the current setup, sampling is restricted to a solvated spherical region centered on the ligand, focusing on local ligand–environment interactions relevant to LIE calculations. Therefore, these simulations were not intended to describe slow global conformational rearrangements of TYR, but to obtain converged averages of ligand–protein and ligand–solvent electrostatic and vdW interaction energies in the bound and unbound states. Recent benchmarking of QligFEP using spherical boundary conditions has shown that focusing simulations on the binding-site region can substantially reduce the number of simulated atoms while preserving the accuracy of ligand free-energy calculations [55]. Although that benchmark specifically addressed FEP rather than LIE, it supports the broader methodological rationale of using localized SCAAS simulations for binding-site energetic calculations. In this study, convergence was assessed directly by monitoring the running averages of the electrostatic and vdW energy components for each replica and for the ensemble average across replicas (Fig. S3, Supporting Information). This protocol was applied to both the free TzD in solution and the TYR–TzD complex in solution for subsequent application of the LIE approach [56, 57]. In the water simulations, ligands were kept centered within their respective simulation spheres by applying a weak harmonic restraint to their center of mass.

## System setup for FEP

For the FEP transformations, the docked pose of T11 was selected as the reference structure. Perturbation pathways between ligands were selected based on maximum common substructure (MCS) analysis, ensuring that each transformation involves minimal structural change, specifically the introduction or modification of a single substituent at either R_1_ or R_3_. Only structurally similar pairs were included because lower structural similarity increases the risk of convergence failure in alchemical FEP calculations [58, 59]. The resulting perturbation network (Fig. 3) was designed to be internally consistent, allowing thermodynamic cycle closure errors to be evaluated as an internal quality-control measure for the FEP results. This mapping ensured that equivalent atoms occupied corresponding positions in space, minimizing artificial strain during alchemical transitions and improving simulation stability [60]. The TzDs listed in Table 2 were analyzed by FEP calculations using the QligFEP protocol [60].

**Table 2:** The subset of TzDs selected for relative binding free energy calculations using QligFEP, based on structural similarity and experimental activity profiles


TzD	*R*_1_	*R*_3_
T01	F	H
T02	F	F
T03	F	Br
T04	F	I
T09	H	H
T10	H	F
T11	H	Br
T12	H	I

The QligFEP protocol involves conducting each ligand transformation in two environments: the protein-bound state, in which the ligand is bound to TYR, and the solvated state, in which the ligand is simulated in water [60]. This approach follows a standard thermodynamic cycle to calculate relative binding free energies from the difference between the protein and water transformation legs. As in the LIE simulations, a spherical solvation boundary with a radius of 20 Å was implemented using the SCAAS model [50], allowing the calculations to focus on the binding-site region rather than the full protein–solvent system. This localized strategy is consistent with recent QligFEP benchmarking, which shows that spherical boundary conditions can substantially reduce system size while retaining competitive accuracy relative to established open-source and commercial FEP protocols [55]. In that benchmark, QligFEP simulations used binding-site-centered spherical systems of approximately 6,000–6,500 atoms and multiple independent replicates, demonstrating the utility of focused simulations for scalable ligand-free-energy calculations. In the present TYR systems, the 20 Å sphere was selected based on previous TYR applications [29–31] and was sufficient to include the ligand, the binuclear Cu²⁺ active site, first-shell interacting residues, and the surrounding solvated environment. Solute atoms located within the outer 3.5 Å layer of the sphere were mildly restrained using a force constant of 10 kcal/(mol·Å²).

In contrast, atoms outside the sphere were tightly constrained (200 kcal/(mol·Å²)) and excluded from the calculations of nonbonded interactions. Long-range interactions beyond a 10 Å cutoff were treated using the LRF method [51], except for atoms involved in FEP transformations, for which no cutoff was applied. The simulation sphere underwent a restrained equilibration phase, during which the temperature was gradually increased from 0.1 to 310 K, while positional restraints on heavy atoms were progressively released from an initial force constant of 50 kcal/(mol·Å^2^). This protocol has been previously employed in TYR systems [31].

In the present study, the relative binding free energy between two ligands, A and B ($\:{\varDelta\:\varDelta\:G}_{bind}$), was calculated using the FEP method in conjunction with the QligFEP package [60]. This tool implements the dual topology paradigm, wherein each perturbation involves the simultaneous appearance of ligand B atoms and disappearance of ligand A atoms, and includes an intermediate step employing soft-core potentials to ensure convergence [61]. Half-harmonic distance restraints of 2.0 kcal/(mol·Å²) were applied to maintain pairs of equivalent (non-dummy) atoms between ligands A and B within a distance window of 0.0–0.2 Å.

FEP calculations are based on the thermodynamic cycle shown in Eq. (1).

1 $$\:{\varDelta\:\varDelta\:G}_{bind}={\varDelta\:G}_{complex}^{A\to\:B}-{\varDelta\:G}_{solvent}^{A\to\:B}$$

The free energy difference for the transformation from ligand A to ligand B ($\:{\varDelta\:G}_{A\to\:B})$ was calculated employing Zwanzig’s exponential formula, as detailed in Eq. (2). In this context, $\:\beta\:$ denotes the inverse thermal energy, $\:\varDelta\:U$ represents the change in effective potential energy between successive FEP windows, and the angular brackets indicate ensemble averaging. The transformation proceeds through a series of intermediate states, with the effective potential described by Eq. (3).

2 $$ \Delta G_{{A \to B}} = - k_{B} T\ln \left\langle {\exp \left( { - \beta \Delta U} \right)} \right\rangle _{A} $$

3 $$\:{U}_{i}=(1-{\lambda\:}_{i}){U}_{A}+{\lambda\:}_{i}{U}_{B}$$

In this framework, $\:\lambda\:$ serves as the coupling parameter that interpolates between the initial state (A) and the final state (B). This parameter was incremented from 0 to 1 using 51 uniformly spaced λ-windows. For each window, sampling was performed for 30 picoseconds (ps) with a time step of 1 femtosecond (fs) across 10 independent MD replicas, which differed only in their initial velocities generated according to the Maxwell–Boltzmann distribution. The use of multiple independent replicas follows the QligFEP strategy, which improves statistical reliability by replicating both the protein-bound and water legs of the thermodynamic cycle. Recent large-scale QligFEP benchmarking employed even shorter per-window sampling with 10 independent replicas per leg, demonstrating that this replicate-based spherical-boundary protocol can provide competitive relative binding free-energy predictions when applied to congeneric ligand transformations [55]. Therefore, the present setup, using 30 ps per λ-window and 10 independent replicas, is consistent with the methodological assumptions of QligFEP and provides a conservative sampling length relative to this recent benchmark.

## LIE calculations

Binding free energies were estimated using data from MD simulations conducted with the Q6 package [42]. These simulations provided interaction energy values for each ligand and its surrounding environment in both bound and unbound states, which were then used in the LIE calculations [56, 57].

Binding free energies of selected TzDs were calculated using the LIE formalism, as follows:

4 $$ \Delta G_{{LIE}} = \alpha \left\langle {V_{{vdW}}^{{bound}} - V_{{vdW}}^{{free}} } \right\rangle + \beta \left\langle {V_{{ele}}^{{bound}} - V_{{ele}}^{{free}} } \right\rangle + \gamma $$

where $\:{V}_{vdW}$ and $\:{V}_{ele}$ represent the differences in average nonpolar (van der Waals) and polar (electrostatic) interaction energies between the ligand and its surroundings in the bound and unbound states, respectively [56]. The alpha, beta, and gamma coefficients are empirical scaling factors applied to the nonpolar, polar, and constant contributions. In the standard LIE model, α is typically set to 0.18, while β varies depending on the chemical nature and polarity of the ligand [57]. In this study, the values used were α = 0.18, β = 0.43, and γ = − 6.16, where the γ coefficient was estimated through linear correlation with the experimental binding free energies (ΔG_EXP_) (Eq. (5)), as applied previously [30].

5 $$\:{\varDelta\:G}_{EXP}=\:RTln{IC}_{50}$$

## Results and discussion

### Preliminary ADMET and molecular docking analysis

We analyzed 48 structurally diverse 1,2,4-triazole derivatives (TzDs) previously reported as TYR inhibitors [25]. These compounds were evaluated against *Agaricus bisporus* TYR, with IC₅₀ values ranging from 4.8 nM (T11) to 15,200 nM (T30), thus covering a broad range for structure–activity relationship (SAR) analysis (see Table 1 in the Methods section). The compounds were classified into five structural groups based on chemical scaffolds and substitution patterns: (1) TzDs with methoxyphenyl or butyl groups at the R₂ position; (2) semicarbazide-TzD hybrids; (3) thiosemicarbazide-TzD hybrids; (4) aryl phenoxymethyl-TzDs conjugated to thiosemicarbazides; and (5) bi-heterocyclic TzDs with triazole and aminothiazole moieties.

Because TYR inhibitors are mainly investigated for topical depigmenting applications, the ADMET analysis was interpreted primarily as an early-stage physicochemical screen for dermal exposure rather than as a direct prediction of clinical or formulation performance. For this purpose, we focused on log Kp as a relative indicator of skin permeability, LogP as a descriptor of lipophilicity relevant to stratum corneum partitioning, TPSA as a proxy for passive permeability, and aqueous solubility as a preliminary indicator of formulation feasibility. Lipinski’s Rule of Five [62] was retained only as a secondary drug-likeness descriptor, since oral bioavailability is not the primary route of administration for most depigmenting TYR inhibitors. Similar descriptor-based ADMET interpretations have been used in recent integrated in silico workflows combining molecular docking, quantum chemical analysis, and pharmacokinetic filtering [63, 64]. Nevertheless, these parameters should be regarded as prioritization criteria, and experimental skin permeation, cytotoxicity, and formulation stability assays remain necessary before any claim of topical suitability can be made. The predicted ADMET properties of all 48 TzDs, obtained with SwissADME [34] are summarized in Table 3.

**Table 3:** Predicted ADME properties of the 48 TzDs obtained using the SwissADME tool

TzD	Log Kp (cm s^−1^)	TPSA (A^2^)	Solubility	LogP	Lipinski	TzD	Log Kp (cm s^−1^)	TPSA (A^2^)	Solubility	LogP	Lipinski
KA	− 7.62	70.67	Soluble	1.12	0	T48	− 7.52	151.54	Soluble	2.85	0
T01	− 5.85	105.34	Moderately	2.28	0	T49	− 7.52	151.54	Soluble	2.73	0
T02	− 5.74	94.34	Moderately	3.05	0	T50	− 7.79	151.54	Soluble	2.55	0
T03	− 5.70	94.34	Poorly	3.27	1	T51	− 7.96	160.77	Soluble	2.95	0
T04	− 6.01	94.34	Poorly	3.29	1	T52	− 7.96	160.77	Soluble	2.81	0
T05	− 5.68	85.11	Moderately	2.62	0	T53	− 7.64	171.34	Soluble	2.40	1
T06	− 5.72	85.11	Moderately	2.89	0	T54	− 7.88	151.54	Soluble	2.55	0
T07	− 5.67	85.11	Moderately	3.19	0	T55	− 7.92	151.54	Soluble	3.00	0
T08	− 5.99	85.11	Moderately	3.19	0	T56	− 7.28	151.54	Moderately	2.91	0
T09	− 5.67	94.34	Moderately	2.89	0	T57	− 7.35	151.54	Soluble	3.19	0
T10	− 5.71	94.34	Moderately	3.03	0	T58	− 7.97	197.36	Soluble	2.30	1
T11	− 5.66	94.34	Poorly	3.46	0	T60	− 6.04	152.26	Moderately	3.24	0
T12	− 5.97	94.34	Poorly	3.32	1	T61	− 6.04	152.26	Moderately	3.21	0
T20	− 6.10	110.05	Moderately	3.99	0	T62	− 6.04	152.26	Moderately	3.02	0
T25	− 6.35	110.05	Moderately	3.15	0	T63	− 6.04	152.26	Moderately	2.97	0
T26	− 6.35	110.05	Moderately	3.20	0	T64	− 6.04	152.26	Moderately	3.08	0
T28	− 6.14	110.05	Moderately	3.28	1	T65	− 6.22	152.26	Moderately	3.08	0
T30	− 6.28	125.07	Moderately	3.14	0	T66	− 5.99	152.26	Moderately	3.05	0
T31	− 6.58	125.07	Moderately	2.50	0	T67	− 5.82	152.26	Moderately	3.41	0
T32	− 6.62	125.07	Moderately	3.31	0	T68	− 5.99	152.26	Moderately	2.93	0
T33	− 6.00	125.07	Moderately	3.51	0	T69	− 6.41	161.49	Moderately	2.95	0
T35	− 6.30	125.07	Moderately	3.53	0	T70	− 6.39	152.26	Moderately	2.35	0
T37	− 6.07	125.07	Moderately	3.54	0	T71	− 6.51	152.26	Moderately	2.71	0
T40	− 6.54	125.07	Moderately	3.36	1	T72	− 6.27	152.26	Moderately	2.42	0
T46	− 7.75	151.54	Soluble	3.05	0	T49	− 7.52	151.54	Soluble	2.73	0

Overall, the ADME results should be interpreted as a preliminary physicochemical filter rather than as evidence of topical efficacy. Most TzDs showed LogP values between 1.1 and 3.9, a range generally compatible with passive diffusion through lipid-rich barriers, and molecular sizes consistent with drug-like chemical space. However, the predicted log Kp values were negative for all compounds, indicating limited intrinsic skin permeability. Within this context, compounds with log Kp values closer to − 5.7, particularly several members of the T01–T12 subset, may be considered comparatively more favorable for topical exposure than analogs with values below − 7.5. TPSA values below 140 Å² further suggest acceptable passive permeability for many compounds, although derivatives with TPSA above this threshold, such as T49–T58 and T69, may require formulation strategies to improve delivery. Therefore, the ADMET analysis alone does not demonstrate topical suitability, but it indicates that several TzDs possess physicochemical properties compatible with further optimization as dermal TYR inhibitors. This interpretation is consistent with recent in silico studies that use ADMET descriptors as early-stage prioritization criteria rather than as substitutes for experimental permeability, cytotoxicity, or formulation assays [63, 64].

Following ADMET analysis, molecular docking was used to assess the binding modes and interaction patterns of TzDs against TYR*Bm*, using KA as the reference inhibitor. Despite its limitations [65], molecular docking remains useful in early-stage structure-based drug design. For TYR systems, the MVD protocol has previously provided plausible binding poses for TYR inhibitors [29–31].

Whereas many previous studies have used *Agaricus bisporus* TYR (TYR*Ab*, PDB ID: 2Y9X) [36], we used *Bacillus megaterium* TYR (TYR*Bm*, PDB ID: 5I38) [35]. TYR*Bm* and TYR*Ab* share approximately 58% sequence identity within a 6 Å radius of the binuclear Cu²⁺ catalytic site [37], indicating conserved coordination geometry and active-site topology. TYR*Bm* also exhibits closer homology to human tyrosinase-related proteins; for instance, human TYRP1 shares 32% sequence identity with TYR*Bm* and exhibits a root-mean-square-deviation (RMSD) of 1.60 Å across 263 aligned residues [4]. This similarity supports the use of TYR*Bm* as a structural model for TYR inhibition. In addition, the structure of TYR*Ab* contains a non-conserved thioether bond between His85 and Cys83, which artificially constrains the copper site [66]. Although this feature may stabilize the active site in TYR*Ab*, it is absent in both TYR*Bm* and human TYR. Its ambiguous role in catalysis further supports our decision to avoid introducing non-representative structural artifacts into our modeling. Bacterial TYR also offers practical advantages over mushroom TYR. Although mushroom TYR is commercially available, it is difficult to engineer genetically and remains challenging to produce recombinantly [67]. In contrast, bacterial TYR can be readily expressed in *E. coli*, which facilitates large-scale production and protein engineering for mechanistic studies [68].

**Fig. 2:**
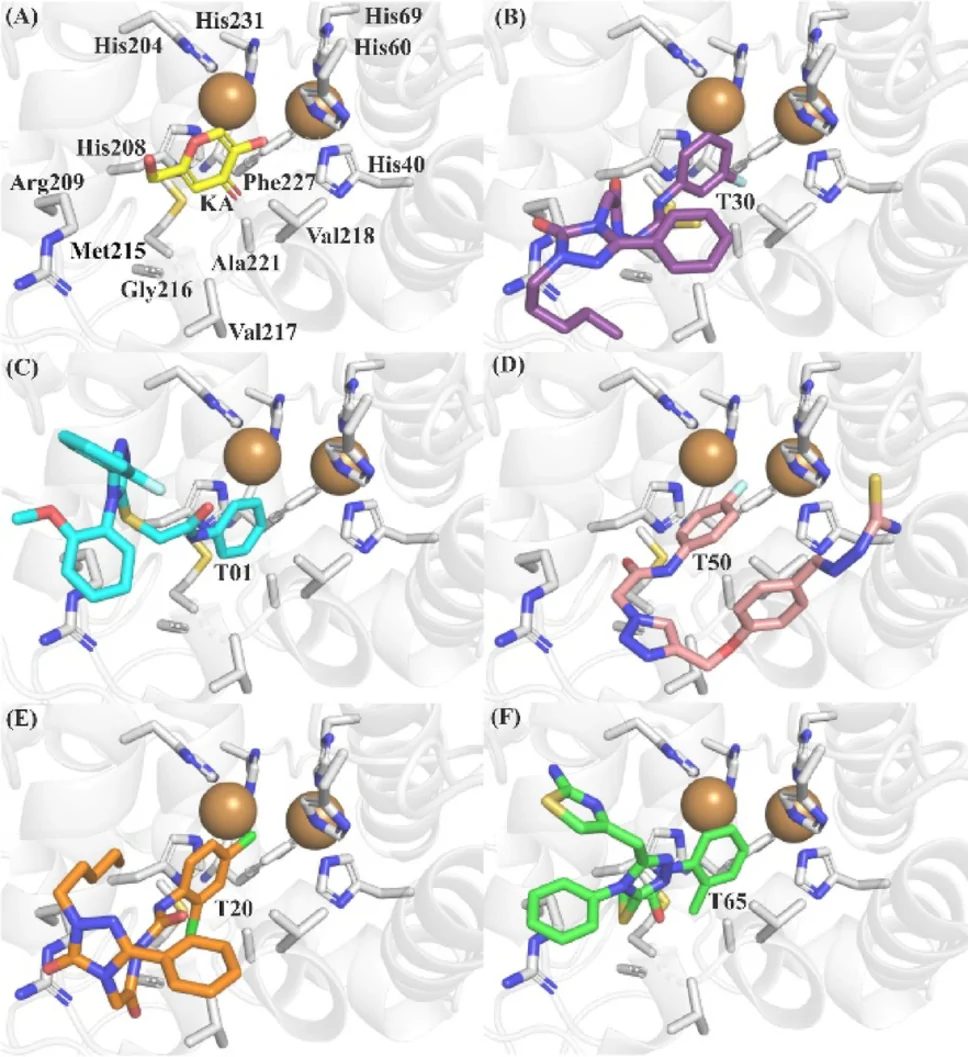
Docking poses of five representative TzDs: **B** T01 (Group 1), **C** T20 (Group 2), **D** T30 (Group 3), **E** T50 (Group 4), and **F** T65 (Group 6). Panel A shows the crystallographic pose of KA from PDB ID 5I38 [35] as a reference. For clarity, residue labels are shown only in panel A, and hydrogen atoms have been omitted. 2D interaction diagram (Fig.e S1) and corresponding PDB files are provided as Supporting Information (SI)

Distinct binding orientations were observed for the different TzD groups within the TYR active site. In group 1 (Fig. 2B), the phenylacetamide moiety orients toward the TYR metal center, closely mimicking the binding mode of KA through its carbonyl and hydroxyl functionalities (Fig. 2A). The halogen substituents within this motif influence binding affinity, likely through electronic effects and steric interactions. In group 2 (Fig. 2C), the phenyl ring of the N-phenyl-2-propionylhydrazine-1-carboxamide moiety is directed toward the metal center, suggesting direct involvement in active-site coordination. A similar orientation is found in group 3 (Fig. 2D), where the N-phenyl-2-propionylhydrazine-1-carbothioamide fragment positions its phenyl group toward the catalytic core, which may enhance metal coordination via the thioamide group. In Group 4 (Fig. 2E), the aromatic ring connected to an N-methylacetamide motif is crucial in interactions with the metal center. For group 5 (Fig. 2F), binding is predominantly driven by the aromatic ring linked to the N-methyl-2-(methylthio)acetamide moiety, which also aligns toward the metal center of TYR. These findings suggest that specific pharmacophoric elements determine the orientation of each group, whereas the remaining substituents mainly form complementary hydrophobic and hydrogen-bond interactions with residues surrounding the active site.

The MOLDOCK [38] scoring function yielded more favorable docking scores for several TzDs than for KA (− 76.12 kcal/mol), indicating more favorable predicted accommodation within the TYR active site (Table 4). T28, T54, and T55 showed the most favorable MOLDOCK scores (− 134.00, − 131.58, and − 130.09 kcal/mol, respectively), suggesting the most favorable predicted binding geometries among the compounds studied. Among the most effective derivatives, a common characteristic was their formation of hydrophobic contacts with a conserved group of residues in the TYR active site, notably Met215, Val217, Val218, Ala221, and Phe227 (Table 4). These residues are known to stabilize inhibitors through hydrophobic and π-π interactions [69–73]. The top-scoring compounds also showed increased hydrophobic interactions, often involving 7–8 distinct residues. This pattern supports the importance of hydrophobic contacts in determining binding strength.

**Table 4:** Docking energy values (MOLDOCK score, kcal/mol), hydrogen bonds (HB), and hydrophobic interactions (HI) for TYR–TzD complexes

TzD	MOLDOCK	HB	HI
KA	− 76.12	-	Met215; Gly216; Val217; Val218; Ala221.
T01	− 71.36	Gly216	Met215; Val217; Val218; Ala221; Phe197.
T02	− 83.54	Gly216	Met215; Val217; Val218; Ala221; Phe197; Phe227.
T03	− 76.38	Gly216	Met215; Val217; Val218; Ala221; Pro201; Phe197; Phe227.
T04	− 85.20	Gly216	Met61; Met215; Val217; Val218; Ala221; Phe197; Phe227.
T05	− 80.65	Gly216	Met215; Val217; Val218; Ala221; Pro201; Phe227.
T06	− 90.41	Gly216	Met215; Val217; Val218; Ala221; Pro201; Phe197; Phe227.
T07	− 82.86	Gly216	Met215; Val217; Val218; Ala221; Phe227.
T08	− 84.20	Gly216	Met215; Val217; Val218; Ala221; Phe227.
T09	− 91.12	Gly216	Met215; Val217; Val218; Ala221; Phe197; Phe227.
T10	− 88.44	Gly216	Met215; Val217; Val218; Ala221; Phe197; Phe227.
T11	− 86.70	Gly216	Met215; Val217; Val218; Ala221; Pro201; Phe227.
T12	− 82.74	Gly216	Met215; Val217; Val218; Ala221; Pro201; Phe227.
T20	− 100.86	-	Met61; Met215; Val217; Val218; Ala221; Pro201.
T25	− 73.38	Gly216	Met61; Met215; Val217; Val218; Ala221; Pro201.
T26	− 125.48	Gly216	Met215; Val217; Val218; Ala221; Pro201, Phe227.
T28	− 134.00	Gly216	Met61; Met215; Val217; Val218; Ala221; Phe197; Phe227; Pro201.
T30	− 124.13	Gly216	Met61; Met215; Val217; Val218; Ala221; Phe227.
T31	− 120.81	-	Met61; Met215; Val217; Val218; Ala221.
T32	− 123.37	Gly216	Met61; Met215; Val217; Val218; Ala221; Phe227; Pro201.
T33	− 113.08	Gly216	Met215; Val217; Val218; Ala221; Phe227.
T35	− 114.57	-	Met61; Met215; Val217; Val218; Ala221; Phe227; Pro201.
T37	− 117.61	-	Met61; Met215; Val217; Val218; Ala221; Phe197; Pro201.
T40	− 112.60	Gly216	Met61; Met215; Val217; Val218; Ala221; Phe197; Pro201.
T46	− 123.20	Gly216	Met61; Met215; Val217; Val218; Ala221; Phe227; Pro219.
T48	− 113.99	-	Met61; Met215; Val217; Val218; Ala221; Phe227.
T49	− 122.72	Gly216	Met61; Met215; Val217; Val218; Ala221; Phe227; Pro219.
T50	− 127.43	Gly216	Met61; Met215; Val217; Val218; Ala221; Phe227; Pro219.
T51	− 122.95	Gly216	Met61; Met215; Val217; Val218; Ala221; Phe227; Pro219.
T52	− 125.10	Gly216	Met61; Met215; Val217; Val218; Ala221; Phe227; Pro219.
T53	− 108.91	Gly216	Met61; Met215; Val217; Val218; Ala221; Phe227; Pro219.
T54	− 131.58	-	Met61; Met215; Val217; Val218; Ala221; Phe227; Pro219.
T55	− 130.09	-	Met61; Met215; Val217; Val218; Ala221; Phe227; Pro219.
T56	− 116.49	-	Met61; Met215; Val217; Val218; Ala221; Phe227; Pro219.
T57	− 112.46	-	Met61; Met215; Val217; Val218; Ala221; Phe227; Pro219.
T58	− 103.40	-	Met61; Met215; Val217; Val218; Ala221; Phe227; Pro219.
T60	− 94.13	-	Met61; Met215; Met184; Val217; Val218; Ala221; Phe197; Phe227.
T61	− 74.63	-	Met61; Met215; Val217; Val218; Ala221.
T62	− 81.96	-	Met61; Met215; Met184; Val217; Val218; Ala221; Phe197; Phe227.
T63	− 97.72	-	Met61; Met215; Met184; Val217; Val218; Ala221; Phe197; Phe227.
T64	− 88.54	-	Met61; Met215; Met184; Val217; Val218; Ala221; Phe197; Phe227.
T65	− 86.14	-	Met61; Met215; Val217; Val218; Ala221; Phe197; Phe227; Pro201.
T66	− 80.24	-	Met61; Met215; Met184; Val217; Val218; Ala221; Phe197; Phe227.
T67	− 84.88	-	Met61; Met215; Met184; Val217; Val218; Ala221; Phe197; Phe227.
T68	− 89.11	-	Met61; Met184; Val217; Val218; Ala221; Phe197; Phe227.
T69	− 93.68	Gly216	Met61; Met184; Met215; Val217; Val218; Ala221; Phe197; Phe227.
T70	− 76.07	Gly216	Met61; Met215; Met184; Val217; Val218; Ala221.
T71	− 85.68	-	Met61; Met215; Met184; Val217; Val218; Ala221; Phe197; Phe227.
T72	–112.22	-	Met61; Met215; Val217; Val218; Ala221; Phe197; Phe227; Pro201.

Moreover, Gly216 was identified as a frequent hydrogen-bonding partner among the most potent inhibitors, such as T28, T50, and T52. This residue likely acts as an anchoring point that helps define ligand orientation within the active site. Notably, although not all leading binders formed explicit hydrogen bonds, strong hydrophobic interactions and favorable binding orientations likely compensated for the lack of polar contacts in some instances (T54 and T55).

In contrast, KA showed a less favorable MOLDOCK score than several triazole derivatives, consistent with its smaller size and fewer hydrophobic contacts under this scoring framework. Aromatic and aliphatic substituents in these ligands may improve binding by enhancing complementarity with the TYR hydrophobic pocket.

These results are consistent with prior studies suggesting that optimizing TYR inhibition can be achieved through hydrogen bonding and hydrophobic interactions [69–73]. The docking results indicate that specific substitutions that strengthen interactions with Phe227 and Pro201, residues not present in the KA interaction profile, can be leveraged to design more effective TYR inhibitors. Overall, the docking analysis identifies the main structural determinants of TYR recognition within the TzD series.

While the MOLDOCK scoring function outputs values in kcal/mol, these scores do not reflect absolute binding free energies and should not be compared directly to experimental affinity values [28]. Instead, they help rank binding poses within triazole derivative series, allowing us to identify plausible orientations and key interactions of each ligand. We then refine these findings using MD simulations and estimate binding free energies using the Linear Interaction Energy (LIE) and Free Energy Perturbation (FEP) methods, which provide a more reliable estimate of binding affinity than docking scores alone [59].

## MD/FEP calculations

Because no crystal structure of a TYR–TRD complex is available, molecular docking was first used to predict plausible binding modes for the TzDs. While docking provides a helpful starting point for modeling protein-ligand interactions, its limitations, including rigid-receptor assumptions and simplified scoring functions [65], require the use of more robust methods for binding-mode evaluation. To address this, MD simulations were performed to account for protein and ligand flexibility, followed by FEP calculations to quantitatively evaluate and validate the binding affinities of the selected triazole derivatives. This workflow improves the reliability of the predicted TYR–ligand complexes [29–31].

For FEP calculations, we selected a subset of triazole derivatives to examine the effects of halogen substitution at R_1_ and R_3_. Selection was based on structural similarity and the availability of experimental IC_50_ data, allowing us to isolate the steric and electronic effects of halogens (F, Br, and I) within a consistent scaffold. This design reduces perturbation complexity and improves convergence. The selected compounds are consistent with the observed SAR trends and experimental potency rankings.

**Fig. 3:**
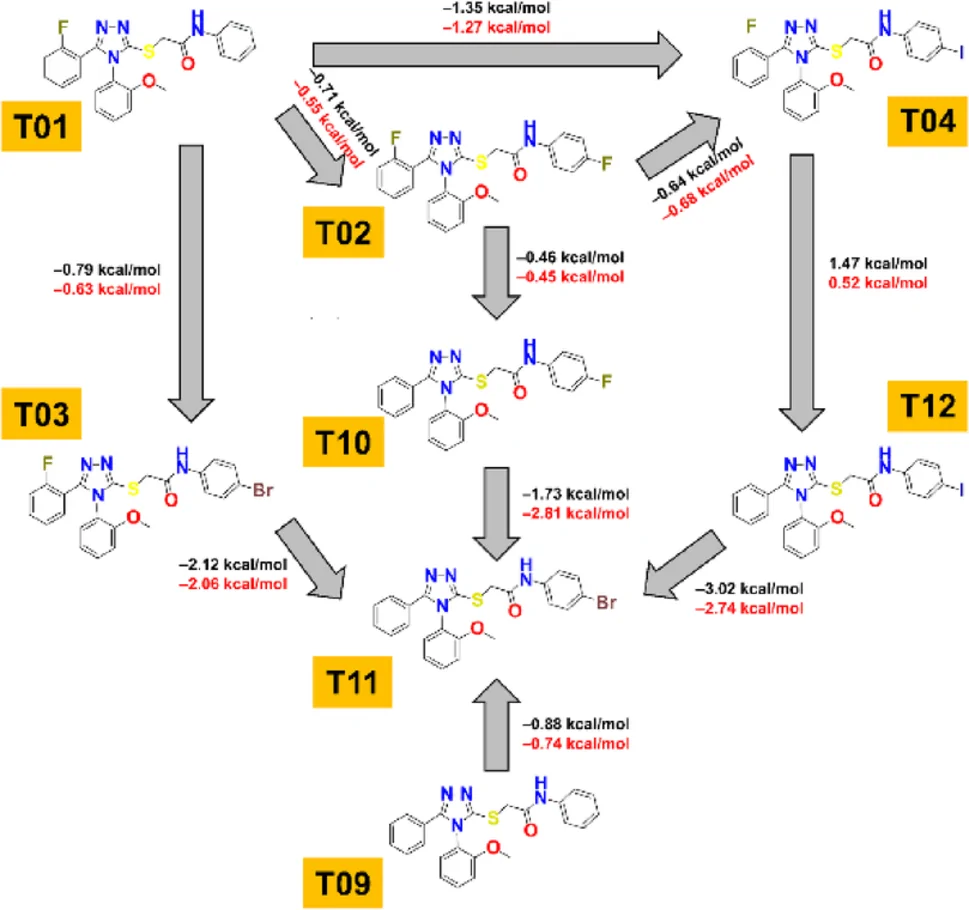
Structural perturbations across a congeneric series of TzDs featuring different substitution patterns. Arrows indicate ligand transformations, with associated relative binding free energy changes (ΔΔG) shown for both experimental values (black) and QligFEP-calculated values (red)

FEP calculations were performed with the QligFEP protocol [60], which uses a dual-topology scheme with soft-core potentials. This protocol calculates relative binding free energies for structurally similar ligands and has been validated across diverse ligand–receptor systems [31, 74]. A total of 10 TzD transformations were selected for MD/FEP analysis (Fig. 3). As shown in Fig. 3, the T11 derivative served as the central reference compound in all computed transformations. Using T11 as the hub enables direct pairwise comparison of relative binding free energies and improves the internal consistency of the perturbation network.

**Table 5:** Calculated and experimental shifts in binding free energies of TzDs as TYR inhibitors. All values are expressed in kcal/mol

TzD Transformation (A → B)	ΔΔG_bind, exp_^a^	ΔΔG_bind, calc_^b^	SEM_f_^c^
T01 → T02	− 0.71	− 0.55	0.10
T01 → T03	− 0.79	− 0.63	0.17
T01 → T04	− 1.35	− 1.27	0.14
T02 → T04	− 0.64	− 0.68	0.29
T02 → T10	− 0.46	− 0.45	0.27
T03 → T11	− 2.12	− 2.06	0.27
T04 → T12	1.47	0.52	0.13
T09 → T11	− 0.88	− 0.74	0.22
T10 → T11	− 1.73	− 2.81	0.34
T12 → T11	− 3.02	− 2.74	0.08

^a^Experimental ∆IC₅₀ values extracted from Vanjare et al. [26] were converted into ∆∆G_bind, exp_ based on the equation $\:{\varDelta\:\varDelta\:G}_{bind,exp}=RTln\:\left(\frac{{IC}_{50}\left(B\right)}{{IC}_{50}\left(A\right)}\right)$

^b^ΔΔG_bind, calc_ values were calculated using the QligFEP package

^c^Associated standard errors of the mean ( $\:SEM=\:\frac{\sigma\:}{\sqrt{n}}$, where σ = standard deviation; *n* = 10 FEP transformations; with the total error, $\:{SEM}_{f}=\:\sqrt{{SEM}_{water}^{2}+{SEM}_{protein}^{2}}\:$)

Relative binding free energies were calculated with an integrated ligand FEP protocol [60] to evaluate the docking-derived binding orientations of the triazole derivatives (Fig. 3 and Table 5), as determined by molecular docking calculations. This approach directly compares relative binding free energies among similar triazole derivatives. A dual-topology scheme was used instead of a conventional single-topology approach. This choice avoids bond formation or breaking within a single-topology framework. Such transformations can introduce numerical instabilities in classical force fields [58, 59].

As shown in Table 5, the relative binding free energies computed with QligFEP agree closely with the experimental measurements (R² = 0.91; MAE = 0.20 kcal/mol; *n* = 10), indicating that the docking-derived binding poses are suitable starting points for free-energy calculations. This agreement supports the use of the docking-derived geometries in the FEP calculations. The linear regression plot for FEP calculations is included in the SI file (Fig. S2).

In the transformations involving T01, T02, T03, and T04, where all compounds possess a F atom at the R_1_ position, inhibitory activity increases progressively when the H atom at R_3_ in T01 is replaced by halogens: F in T02, Br in T03, and I in T04. This trend likely reflects the increasing vdW radii and polarizabilities of the halogens, which enhance dispersive interactions and promote shape complementarity near the Cu(II) ions in the TYR active site. The direct T01→T04 transformation yielded a relative binding free-energy shift of − 1.35 kcal/mol experimentally and − 1.27 kcal/mol computationally. Using T02 as an intermediate, the stepwise transformations T01→T02 and T02→T04 yield a cumulative computed ΔΔG of − 1.23 kcal/mol, close to the direct T01→T04 value and consistent with the internal coherence of the FEP model. Overall, the calculated free-energy shifts reproduce the experimental trends across these transformations.

In the second group (T09, T10, T11, and T12), the H atom at the R_1_ position is retained, while the H at the R_3_ position (T09) undergoes the same series of substitutions as in the previous set: F in T10, Br in T11, and I in T12. Figure 3 shows that the transformations T02→T10, T03→T11, and T04→T12 model the replacement of the F atom at R_1_ by H while preserving the halogen at R_3_. The first two transformations exhibit enhanced TYR inhibitory activity (− 0.45 ± 0.27 and − 2.06 ± 0.27 kcal/mol, respectively), whereas the third displays the only positive shift (0.52 ± 0.13 kcal/mol) in binding free energy within this series. The calculated free-energy shifts for all three transformations are in good agreement with the experimental values.

In the largest transformation, T12→T11, the experimental ΔΔG_bind_ value is − 3.02 kcal/mol, indicating a marked gain in binding affinity. The FEP calculation yielded a ΔΔG_bind_ of − 2.74 ± 0.08 kcal/mol, in close agreement with the experimental value. In a previous application of this methodology to the TYR system, we successfully reproduced experimental data describing the binding modes of arylpiperidine and arylpiperazine derivatives [31]. These results further support the use of QligFEP to evaluate the binding modes of TzDs as TYR inhibitors.

### LIE and residue decomposition analysis

After validating the docking-derived binding mode using FEP calculations on a congeneric halogenated subset, we used the LIE method to extend the binding free-energy analysis to a broader set of TzDs. LIE was selected because it provides absolute binding free-energy estimates and separates electrostatic and vdW contributions, allowing direct interpretation of the energetic factors that distinguish low-, moderate-, and high-activity inhibitors. This method has previously been applied to TYR systems [29–31, 33].

As described in the Methods section, LIE calculations were performed for 15 TYR–TzD systems selected from the original set of 48 compounds (Table 1). The systems were classified into three activity categories based on their experimental activity levels: low activity (T30, T33, T71, T51, and T01), moderate activity (T05, T70, T68, and T63), and high activity (T72, T65, T31, T28, T26, and T20). This stratification allowed evaluation of the model across a broad potency range, from nanomolar to micromolar inhibitors.

Binding free energies were estimated using LIE from 10 independent 2 ns replicas for each TYR–TzD complex and ligand-in-water simulation. We assessed convergence by monitoring running averages of ligand–environment electrostatic and vdW energies. Both energy components quickly reached stable values, with differences between replicas falling within standard errors (see Fig. S3), indicating sufficient sampling for LIE calculations under Q6/SCAAS. QligFEP benchmarking further supports the use of binding-site-focused spherical-boundary simulations for ligand-free-energy calculations [55]. These simulations do not capture slow conformational changes or long-range protein motions, so our results focus on local binding energetics and residue-level interactions in the TYR active site. The empirical parameters α and β were taken from the literature (0.18 and 0.43, respectively), whereas γ was fitted against the experimental free energies, yielding γ = −6.16.

**Fig. 4:**
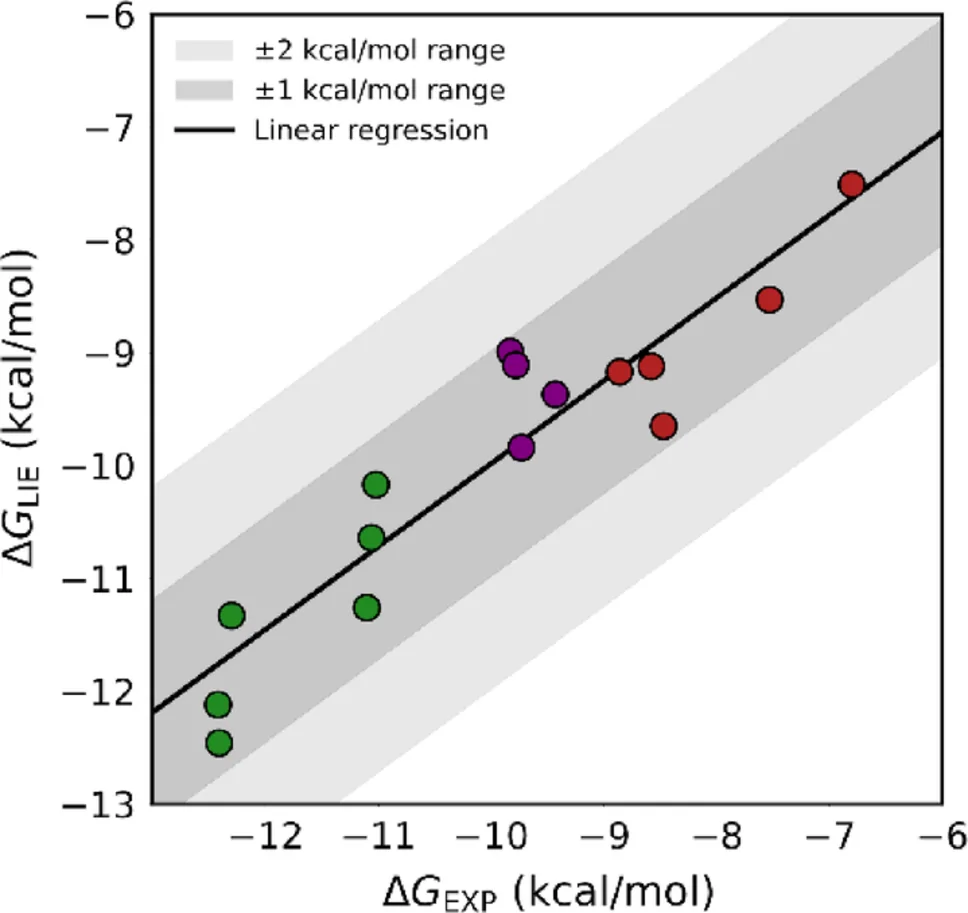
Linear regression model correlating calculated binding free energies (ΔG_LIE_) with experimental values (ΔG_EXP_) for selected TYR-TzD systems. Selected TzDs are categorized by experimental activity: low (red), moderate (purple), and high (green). All values are reported in kcal/mol

The resulting LIE model (Fig. 4) reproduces the absolute binding free energies of the TzDs with good accuracy (R² = 0.93; MAE = 0.54 kcal/mol; *n* = 15). In agreement with our previous findings [30, 31], *vdW* interactions emerged as key contributors to ligand binding. In this study, *vdW* interaction energies ranged from − 27.95 kcal/mol (T70) to − 4.52 kcal/mol (T30), whereas *ele* contributions varied from − 8.35 kcal/mol (T26) to + 4.49 kcal/mol (T68). As shown in Table 6, inhibitors with moderate to high experimental activity consistently displayed lower vdW energies, consistent with our previous studies.

**Table 6:** Calculated (ΔG_LIE_) and experimental (ΔG_EXP_) binding free energies for TzDs as TYR inhibitors. All values are reported in kcal/mol

TzD	$\:{V}_{vdw}^{free}$	$\:{V}_{ele}^{free}$	$\:{V}_{vdw}^{bound}$	$\:{V}_{ele}^{bound}$	$\:{\varDelta\:V}_{vdw}$	$\:{\varDelta\:V}_{ele}$	$\:{\varDelta\:G}_{LIE}$	$\:{\varDelta\:G}_{EXP}$
T20	− 43.07 ± 0.14	− 51.53 ± 0.25	− 69.23 ± 0.66	− 54.45 ± 0.62	− 26.16 ± 0.67	− 2.91 ± 0.66	− 12.12 ± 0.13	− 12.42
T26	− 41.61 ± 0.12	− 54.49 ± 0.20	− 56.61 ± 0.76	− 62.85 ± 1.53	− 14.99 ± 0.77	− 8.35 ± 1.54	− 12.46 ± 0.64	− 12.41
T28	− 43.53 ± 0.20	− 57.56 ± 0.46	− 61.95 ± 1.84	− 61.89 ± 1.29	− 18.41 ± 1.85	− 4.32 ± 1.37	− 11.33 ± 0.25	− 12.30
T31	− 40.47 ± 0.28	− 57.89 ± 0.83	− 64.30 ± 0.18	− 59.78 ± 0.43	− 23.82 ± 0.33	− 1.89 ± 0.93	− 11.26 ± 0.67	− 11.10
T65	− 34.78 ± 0.26	− 48.64 ± 1.02	− 58.96 ± 3.31	− 48.93 ± 2.43	− 24.17 ± 3.32	− 0.29 ± 2.64	− 10.64 ± 1.21	− 11.06
T72	− 36.00 ± 0.37	− 48.73 ± 1.12	− 57.89 ± 1.78	− 48.90 ± 2.00	− 21.88 ± 1.82	− 0.17 ± 2.30	− 10.17 ± 1.18	− 11.02
T63	− 35.57 ± 0.49	− 49.75 ± 0.63	− 50.86 ± 3.75	− 49.93 ± 1.76	− 15.29 ± 3.78	− 0.18 ± 1.87	− 8.99 ± 1.05	− 9.83
T68	− 35.77 ± 0.85	− 48.66 ± 1.25	− 62.93 ± 4.67	− 44.16 ± 3.05	− 27.15 ± 4.74	4.49 ± 3.30	− 9.11 ± 0.61	− 9.78
T70	− 32.84 ± 0.46	− 48.52 ± 0.78	− 60.80 ± 3.52	− 45.39 ± 3.31	− 27.95 ± 3.55	3.12 ± 3.40	− 9.84 ± 0.81	− 9.73
T05	− 32.52 ± 0.25	− 35.11 ± 0.45	− 44.01 ± 3.27	− 37.79 ± 3.38	− 11.48 ± 3.28	− 2.67 ± 3.41	− 9.37 ± 1.17	− 9.43
T01	− 34.41 ± 0.45	− 37.64 ± 0.82	− 56.98 ± 2.44	− 35.21 ± 0.59	− 22.56 ± 2.48	2.42 ± 1.01	− 9.17 ± 0.24	− 8.86
T51	− 38.95 ± 1.01	− 79.77 ± 1.11	− 55.45 ± 3.49	− 79.76 ± 3.22	− 16.49 ± 3.62	0.02 ± 3.40	− 9.12 ± 0.76	− 8.58
T71	− 34.32 ± 0.29	− 48.28 ± 0.57	− 53.33 ± 3.93	− 48.46 ± 2.78	− 19.00 ± 3.94	− 0.17 ± 2.84	− 9.65 ± 1.40	− 8.47
T33	− 43.19 ± 0.17	− 50.98 ± 0.43	− 51.61 ± 1.23	− 52.96 ± 1.26	− 8.42 ± 1.24	− 1.99 ± 1.34	− 8.53 ± 0.57	− 7.53
T30	− 41.67 ± 0.15	− 51.81 ± 0.30	− 46.20 ± 1.66	− 53.07 ± 2.68	− 4.52 ± 1.67	− 1.26 ± 2.70	− 7.51 ± 1.49	− 6.80

The following analysis focuses on three representative inhibitors with distinct activity profiles: T30 (low activity), T05 (moderate activity), and T20 (high activity). This comparison highlights the molecular features underlying their distinct binding behaviors. Notably, the predicted LIE binding free energy (ΔG_LIE_) for T30 underestimates the experimental value by approximately 0.71 kcal/mol (− 6.48 kcal/mol). In contrast, T05 slightly overestimates the experimental free energy by 0.06 kcal/mol (− 9.43 kcal/mol), while the ΔG_LIE_ for T20 overestimates its experimental value by approximately 0.30 kcal/mol (− 12.42 kcal/mol). These deviations fall within acceptable limits and support the model’s predictive reliability.

Although free energy cannot be rigorously decomposed into per-residue contributions from MD simulations [75], residue decomposition was used here to estimate the energetic roles of amino acids near the TYR catalytic site. This analysis considered *vdW* and *ele* components defined by the LIE framework. Residue decomposition helps identify the interactions that contribute most to ligand binding [76]. It also provides a useful framework for interpreting affinity trends and guiding inhibitor optimization.

**Fig. 5:**
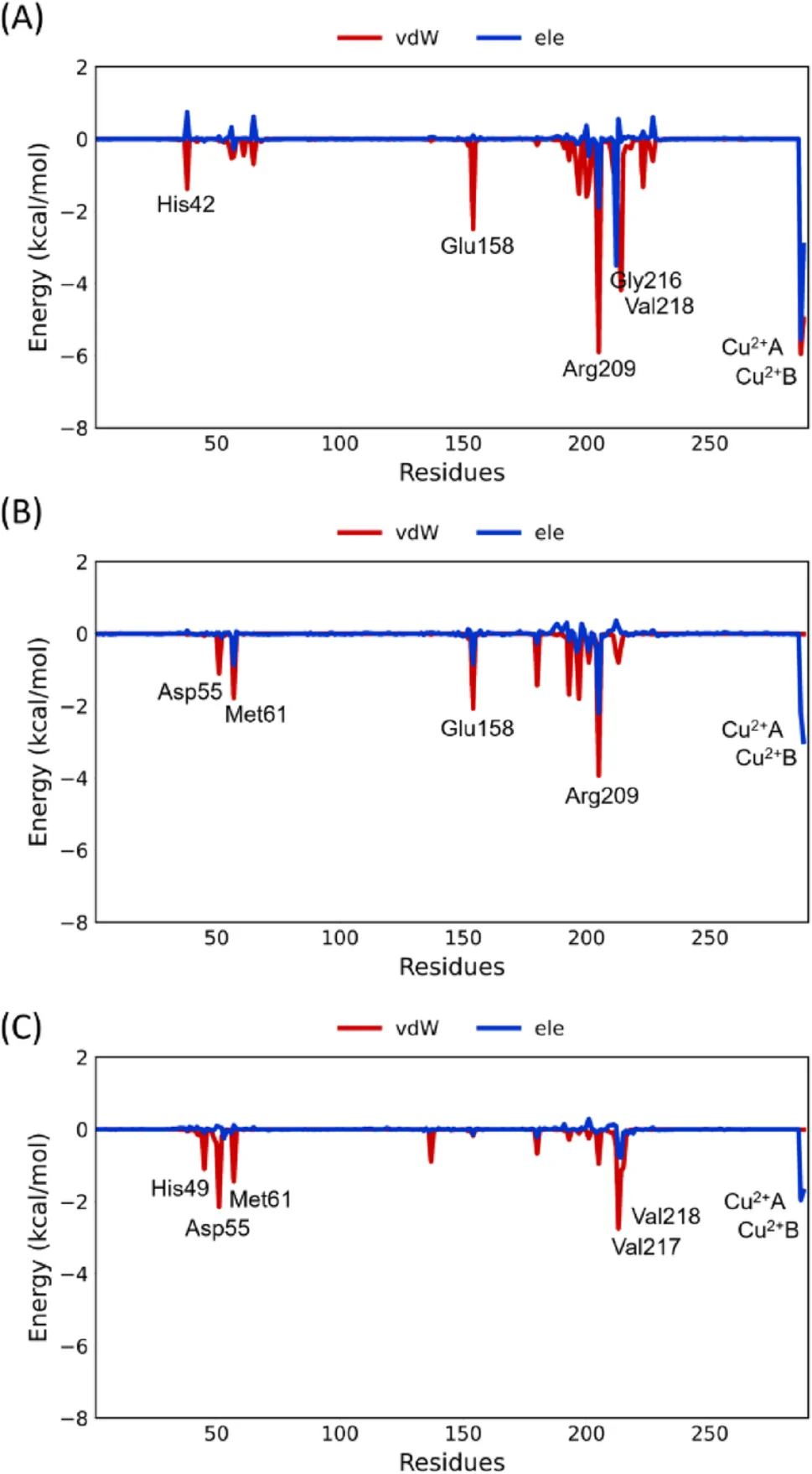
Per-residue van der Waals (red) and electrostatic (blue) energy contributions (in kcal/mol) for selected TYR–TzD complexes included in the LIE analysis. Panels A, B, and C correspond to inhibitors T20 (high activity), T05 (moderate activity), and T30 (low activity), respectively

Figure 5 shows the average vdW (red) and electrostatic (blue) interaction energies for TYR residues that contribute most to ΔG_LIE_. When comparing the high-activity inhibitor T20 with the moderate-activity inhibitor T05, notable differences emerge. In the T05 system, vdW interactions with Gly216, Val217, and Val218 are markedly reduced, shifting from − 1.25 to − 0.57 kcal/mol, − 2.49 to − 0.80 kcal/mol, and − 4.18 to − 0.35 kcal/mol, respectively. Arg209 also exhibits a substantial decrease in interaction energy, from − 5.90 to − 3.93 kcal/mol. These reductions likely contribute to T05’s lower binding affinity.

Comparison of T20 and T30 reveals even larger differences in vdW interactions. For T30, the vdW interaction with Gly216 decreases from − 1.25 to − 0.50 kcal/mol, while the interaction with Val218 reduces from − 4.18 to − 1.14 kcal/mol. The largest change occurs for Arg209, where the interaction decreases from − 5.90 to − 0.95 kcal/mol. This residue is important for accommodating bulky ligands within the TYR active site [35, 77] and has been implicated in TYR catalytic activity [78, 79]. Interactions with the Cu²⁺ ions are also weakened in T30: vdW contributions decrease from − 10.93 to − 0.02 kcal/mol, and electrostatic contributions decrease from − 8.48 to − 3.67 kcal/mol, consistent with its reduced binding affinity.

**Fig. 6:**
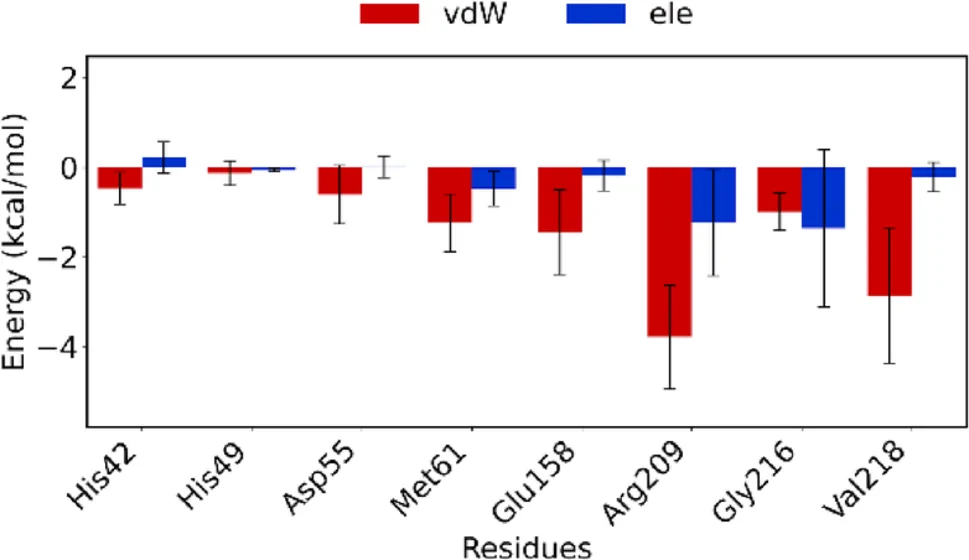
Average van der Waals (red) and electrostatic (blue) interaction energies between TYR active-site residues and 15 TzDs. Bars represent mean interaction energies, and error bars indicate the standard deviation across all complexes. All energy values are reported in kcal/mol. Detailed numerical values are provided in Table S3

As shown in Fig. 6, Arg209, Gly216, and Val218 are among the most influential residues across the TYR–TzD systems, providing strong vdW contributions in the most potent inhibitors, such as T20, T26, T28, and T65. Arg209 contributes up to − 5.90 kcal/mol in T20, while Gly216 and Val218 contribute up to − 1.43 and − 4.39 kcal/mol, respectively, in T65. Together, these residues define a conserved hydrophobic region that stabilizes aromatic or halogenated moieties in high-affinity TzDs. In contrast, compounds with low to moderate activity, including T30, T33, and T05, show weaker interactions with these residues and rely more on less conserved contacts, such as Asp55 or His49, which contribute less consistently across the series.

Electrostatic interactions from residues such as Arg209 and Gly216 also correlate with observed binding trends. In potent inhibitors, including T20, T31, and T28, these residues exhibit moderately stabilizing electrostatic energies, with Arg209 ranging from − 1.89 to − 3.82 kcal/mol. These stabilizing interactions are absent or much weaker in low-affinity ligands. Met61 and Glu158 also make favorable, although more variable, vdW contributions. Overall, these residue-level trends are consistent with the observed SAR and show that efficient engagement of the Arg209–Gly216–Val218 region is associated with stronger binding affinity. These findings support prioritizing substituent patterns that enhance interactions in this region during future ligand optimization.

It is important to clarify the scope of the MD simulations used in this study. The LIE and FEP protocols were designed to sample local ligand–environment interactions within a spherical SCAAS boundary, rather than to explore global TYR dynamics. Therefore, the simulations are suitable for comparing binding energetics among related TzDs and for identifying dominant active-site interactions, but they should not be interpreted as exhaustive descriptions of long-timescale enzyme conformational behavior. Longer periodic-boundary simulations would be more appropriate to investigate large-scale loop rearrangements, allosteric effects, or ligand-induced conformational transitions. In the present study, confidence in the energetic analysis derives from convergence of vdW and electrostatic interaction terms across independent replicas, agreement with experimental affinity trends, consistency between FEP and LIE results, and recent benchmarking showing that QligFEP with spherical boundary conditions can reproduce ligand relative binding free energies with competitive accuracy while focusing the simulation on the binding-site region [55].

### Structural basis of triazole-based TYR inhibition

Taken together, the results from molecular docking, FEP, LIE, and residue decomposition converge on a consistent mechanistic picture of TzD inhibition of TYR*Bm*. Across all computational approaches, vdW interactions emerge as the dominant energetic driver of binding affinity, outweighing electrostatic and hydrogen-bonding contributions. This is consistent with the largely hydrophobic character of the TYR active site, as noted in structural and computational studies of diverse inhibitor classes [35, 71, 73].

The three residues most consistently identified by residue decomposition, Arg209, Gly216, and Val218, collectively constitute the hydrophobic anchorage triad of TYR*Bm*. Arg209, in particular, contributes not only hydrophobic contacts but also electrostatic stabilization in high-affinity inhibitors (e.g., − 3.82 kcal/mol in T28), suggesting a dual role as both an anchorage and selectivity determinant, consistent with prior structural analyses of TYR*Bm* [77, 78]. In contrast, lower-affinity compounds such as T30, T33, and T05 rely predominantly on contacts with peripheral, less conserved residues (Asp55, His49), yielding smaller and less consistent energetic contributions, a pattern also observed for chalcone and coumarin-based TYR inhibitors [69].

A key distinction from prior computational studies of triazole-TYR inhibition is the integration of FEP and LIE into a single framework. While recent experimental studies have advanced TYR inhibitor design through synthesis and biological evaluation of novel hybrid scaffolds [16], previous investigations have relied primarily on docking scores or MM-GBSA estimates [22, 24, 26], which can rank compounds inconsistently relative to experimental IC_50_ values. The predictive accuracy achieved here by FEP (R² = 0.91, MAE = 0.20 kcal/mol) and LIE (R² = 0.93, MAE = 0.54 kcal/mol) validates the use of these higher-level methods for discriminating potency within a congeneric TzD series and establishes a benchmark for future virtual screening campaigns targeting TYR.

From a structure–activity perspective, progressive halogen substitution at R_3_ (H → F → Br → I) consistently improves binding affinity, attributable to the increasing vdW radius and polarizability of larger halogens, which strengthen dispersive contacts within the Cu²⁺-flanking hydrophobic pocket. However, combining F at R_1_ with I at R_3_ (T04 vs. T12) does not follow a simple additive trend, suggesting steric context-dependence that precludes straightforward additivity assumptions. In practical terms, single-site halogenation is a reliable affinity-enhancing strategy, whereas dual-site halogenation requires case-by-case evaluation, preferably via FEP. These findings are consistent with recent experimental observations on halogenated triazole scaffolds as TYR inhibitors [20, 27].

## Conclusions

This study presents an integrated computational framework for the mechanistic characterization of 1,2,4-triazole derivatives as TYR inhibitors, combining ADMET profiling, molecular docking, MD simulations, and binding free-energy calculations based on FEP and LIE. The results indicate that triazole binding to Bacillus megaterium TYR is driven predominantly by van der Waals interactions involving Arg209, Gly216, and Val218, whereas low-affinity analogues rely more on peripheral and less conserved contacts. The FEP-derived binding free energies (R² = 0.91; MAE = 0.20 kcal/mol) and the LIE model (R² = 0.93; MAE = 0.54 kcal/mol) reproduced the experimental inhibitory data with good accuracy, supporting the use of the QligFEP–Q6 workflow to discriminate TYR inhibitor potency within a congeneric series. FEP analysis also indicates that halogen substitution at R₃ enhances binding in a size-dependent manner (F < Br < I), whereas dual halogenation at R₁ and R₃ does not follow a simple additive trend.

From an ADMET perspective, several triazole derivatives showed physicochemical profiles compatible with preliminary prioritization for dermal applications, although experimental skin permeation, cytotoxicity, and formulation stability assays are still required before topical suitability can be established. Among the five structural groups assessed, derivatives bearing semicarbazide or thiosemicarbazide moieties showed a favorable balance between predicted physicochemical properties and binding affinity. Overall, these results suggest that aromatic and halogenated substituents that maximize vdW contacts in the Arg209–Gly216–Val218 region represent useful structural features for the rational optimization of TYR inhibitors. The conclusions of this study are therefore strongest at the level of local active-site binding energetics and SAR interpretation, rather than at the global level of TYR conformational dynamics.

## Supplementary Information

Below is the link to the electronic supplementary material.

Supplementary Material 1

## Data Availability

No datasets were generated or analysed during the current study.
