# Supplementary material for: Rational design of triazole tyrosinase inhibitors via integrated free energy calculations
Source: Mol Divers. 2026 May 29;30(5):8461–78. doi: 10.1007/s11030-026-11601-7 (PMC13635027; doi:10.1007/s11030-026-11601-7)
Supplement: Supplementary file 1 [file 11030_2026_11601_MOESM1_ESM.pdf]

Rational Design of Triazole Tyrosinase Inhibitors via Integrated Free Energy Calculations

Supplementary Information

Table S1. SMILES codes for 48 selected TzDs.

TzD	SMILES code
**T01**	COC1=CC=CC=C1N1C(SCC(=O)NC2=CC=CC=C2)=NN=C1C1=C(F)C=CC=C1
**T02**	COC1=CC=CC=C1N1C(SCC(=O)NC2=CC=C(F)C=C2)=NN=C1C1=C(F)C=CC=C1
**T03**	COC1=CC=CC=C1N1C(SCC(=O)NC2=CC=C(Br)C=C2)=NN=C1C1=C(F)C=CC=C1
**T04**	COC1=CC=CC=C1N1C(SCC(=O)NC2=CC=C(I)C=C2)=NN=C1C1=C(F)C=CC=C1
**T05**	CCCCN1C(SCC(=O)NC2=CC=CC=C2)=NN=C1C1=CC=CC=C1
**T06**	CCCCN1C(SCC(=O)NC2=CC=C(F)C=C2)=NN=C1C1=CC=CC=C1
**T07**	CCCCN1C(SCC(=O)NC2=CC=C(Br)C=C2)=NN=C1C1=CC=CC=C1
**T08**	CCCCN1C(SCC(=O)NC2=CC=C(I)C=C2)=NN=C1C1=CC=CC=C1
**T09**	COC1=CC=CC=C1N1C(SCC(=O)NC2=CC=CC=C2)=NN=C1C1=CC=CC=C1
**T10**	COC1=CC=CC=C1N1C(SCC(=O)NC2=CC=C(F)C=C2)=NN=C1C1=CC=CC=C1
**T11**	COC1=CC=CC=C1N1C(SCC(=O)NC2=CC=C(Br)C=C2)=NN=C1C1=CC=CC=C1
**T12**	COC1=CC=CC=C1N1C(SCC(=O)NC2=CC=C(I)C=C2)=NN=C1C1=CC=CC=C1
**T20**	CCCCCN1N=C(N(CC(=O)NNC(=O)NC2=CC=C(Cl)C=C2Cl)C1=O)C1=CC=CC=C1
**T25**	CCCCCN1N=C(N(CC(=O)NNC(=O)NC2=CC=CC=C2C(F)(F)F)C1=O)C1=CC=CC=C1
**T26**	CCCCCN1N=C(N(CC(=O)NNC(=O)NC2=CC=CC(=C2)C(F)(F)F)C1=O)C1=CC=CC=C1
**T28**	CCCCCN1N=C(N(CC(=O)NNC(=O)NC2=CC(=CC(=C2)C(F)(F)F)C(F)(F)F)C1=O)C1=CC=CC=C1
**T30**	CCCCCN1N=C(N(CC(=O)NNC(=S)NC2=CC=CC(F)=C2)C1=O)C1=CC=CC=C1
**T31**	CCCCN1N=C(N(CC(=O)NNC(=S)NC2=CC=C(F)C=C2)C1=O)C1=CC=CC=C1
**T32**	CCCCN1N=C(N(CC(=O)NNC(=S)NC2=C(F)C=C(F)C=C2)C1=O)C1=CC=CC=C1
**T33**	CCCCCN1N=C(N(CC(=O)NNC(=S)NC2=CC=CC=C2Cl)C1=O)C1=CC=CC=C1
**T35**	CCCCN1N=C(N(CC(=O)NNC(=S)NC2=CC=C(Cl)C=C2)C1=O)C1=CC=CC=C1
**T37**	CCCCN1N=C(N(CC(=O)NNC(=S)NC2=CC(Cl)=CC(Cl)=C2)C1=O)C1=CC=CC=C1
**T40**	CCCCN1N=C(N(CC(=O)NNC(=S)NC2=CC=C(Br)C=C2)C1=O)C1=CC=CC=C1

**T46**	NC(=S)N\N=C\C1=CC=C(OCC2=CN(CC(=O)NC3=CC(Br)=CC=C3)N=N2)C=C1
**T48**	NC(=S)N\N=C\C1=CC=C(OCC2=CN(CC(=O)NC3=CC(Cl)=CC=C3)N=N2)C=C1
**T49**	NC(=S)N\N=C\C1=CC=C(OCC2=CN(CC(=O)NC3=CC=C(Cl)C=C3)N=N2)C=C1
**T50**	NC(=S)N\N=C\C1=CC=C(OCC2=CN(CC(=O)NC3=CC=C(F)C=C3)N=N2)C=C1
**T51**	COC1=CC=CC(NC(=O)CN2C=C(COC3=CC=C(\C=N\NC(N)=S)C=C3)N=N2)=C1
**T52**	COC1=CC=C(NC(=O)CN2C=C(COC3=CC=C(\C=N\NC(N)=S)C=C3)N=N2)C=C1
**T53**	C(=S)N\N=C\C1=CC=C(OCC2=CN(CC(=O)NC3=CC=C(C=C3)[N+]([O-])=O)N=N2)C=C1
**T54**	NC(=S)N\N=C\C1=CC=C(OCC2=CN(CC(=O)NCC3=CC=CC=C3)N=N2)C=C1
**T55**	NC(=S)N\N=C\C1=CC=C(OCC2=CN(CC(=O)NCC3=CC=C(F)C=C3)N=N2)C=C1
**T56**	NC(=S)N\N=C\C1=CC=C(OCC2=CN(CC(=O)NC3=C(Cl)C(Cl)=CC=C3)N=N2)C=C1
**T57**	CC1=C(NC(=O)CN2C=C(COC3=CC=C(\C=N\NC(N)=S)C=C3)N=N2)C=CC=C1Cl
**T58**	CC1=C(NC(=O)CN2C=C(COC3=CC=C(\C=N\NC(N)=S)C=C3)N=N2)C=CC=C1[N+]([O-])=O
**T60**	CC1=CC(C)=C(NC(=O)CSC2=NN=C(CC3=CSC(N)=N3)N2C2=CC=CC=C2)C=C1
**T61**	CC1=CC(NC(=O)CSC2=NN=C(CC3=CSC(N)=N3)N2C2=CC=CC=C2)=C(C)C=C1
**T62**	CC1=CC=CC(C)=C1NC(=O)CSC1=NN=C(CC2=CSC(N)=N2)N1C1=CC=CC=C1
**T63**	CC1=C(C)C=C(NC(=O)CSC2=NN=C(CC3=CSC(N)=N3)N2C2=CC=CC=C2)C=C1
**T64**	CC1=CC(NC(=O)CSC2=NN=C(CC3=CSC(N)=N3)N2C2=CC=CC=C2)=CC(C)=C1
**T65**	CC1=C(NC(=O)CSC2=NN=C(CC3=CSC(N)=N3)N2C2=CC=CC=C2)C=CC=C1
**T66**	CCC1=C(NC(=O)CSC2=NN=C(CC3=CSC(N)=N3)N2C2=CC=CC=C2)C=CC=C1
**T67**	CCC1=C(NC(=O)CSC2=NN=C(CC3=CSC(N)=N3)N2C2=CC=CC=C2)C(C)=CC=C1
**T68**	CCC1=CC=C(NC(=O)CSC2=NN=C(CC3=CSC(N)=N3)N2C2=CC=CC=C2)C=C1
**T69**	CCOC1=CC=C(NC(=O)CSC2=NN=C(CC3=CSC(N)=N3)N2C2=CC=CC=C2)C=C1
**T70**	NC1=NC(CC2=NN=C(SCC(=O)NC3=CC=CC=C3)N2C2=CC=CC=C2)=CS1
**T71**	NC1=NC(CC2=NN=C(SCC(=O)NCC3=CC=CC=C3)N2C2=CC=CC=C2)=CS1
**T72**	NC1=NC(CC2=NN=C(SCC(=O)NCCC3=CC=CC=C3)N2C2=CC=CC=C2)=CS1

Figure S1. 2D interaction diagram for docking poses of five representative TzDs: B) T01 (Group 1), C) T20 (Group 2), D) T30 (Group 3), E) T50 (Group 4), and F) T65 (Group 6)

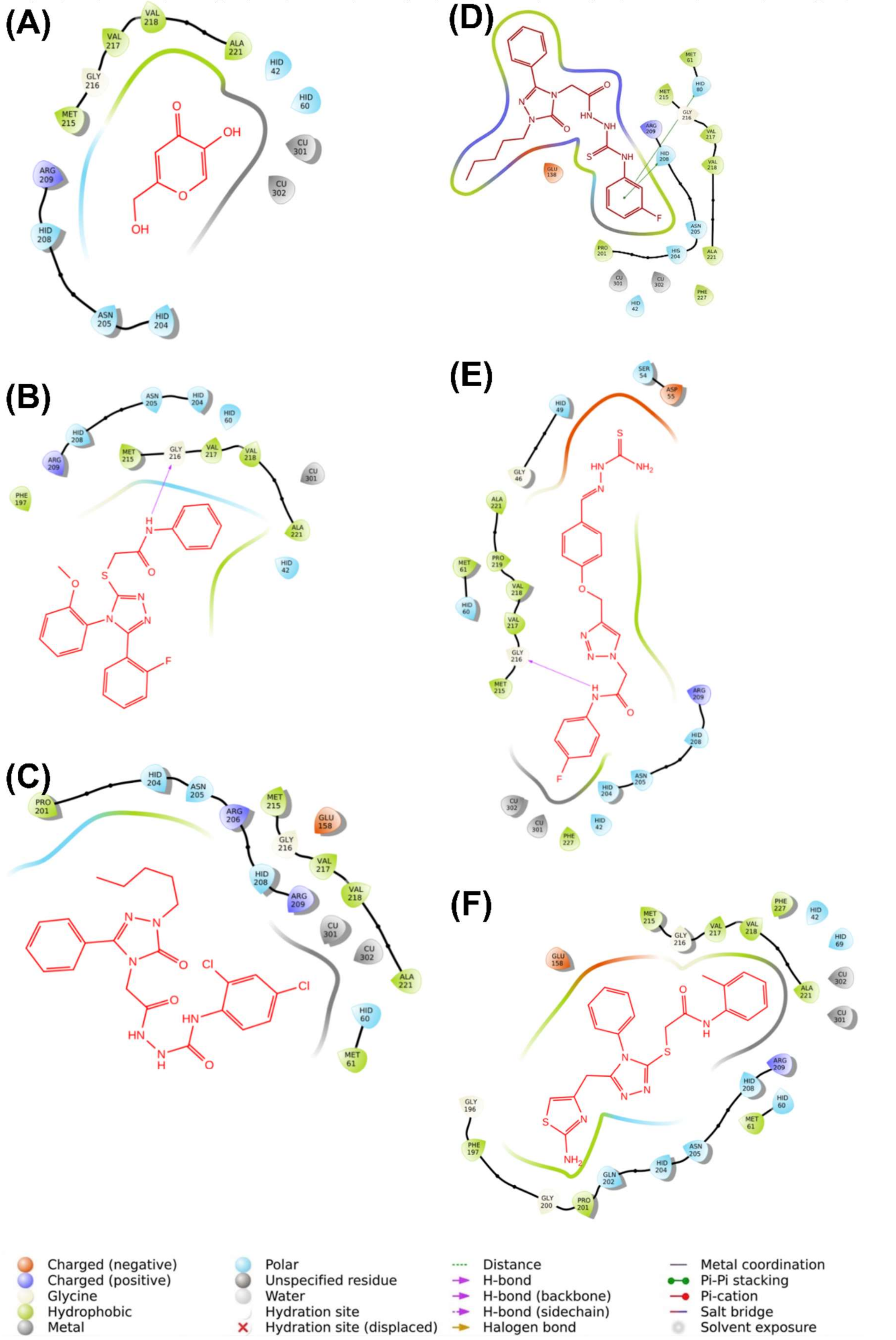

Figure S2. Linear regression model correlating $\Delta\Delta G_{bind,exp}$ and $\Delta\Delta G_{bind,calc}$ from FEP calculations.

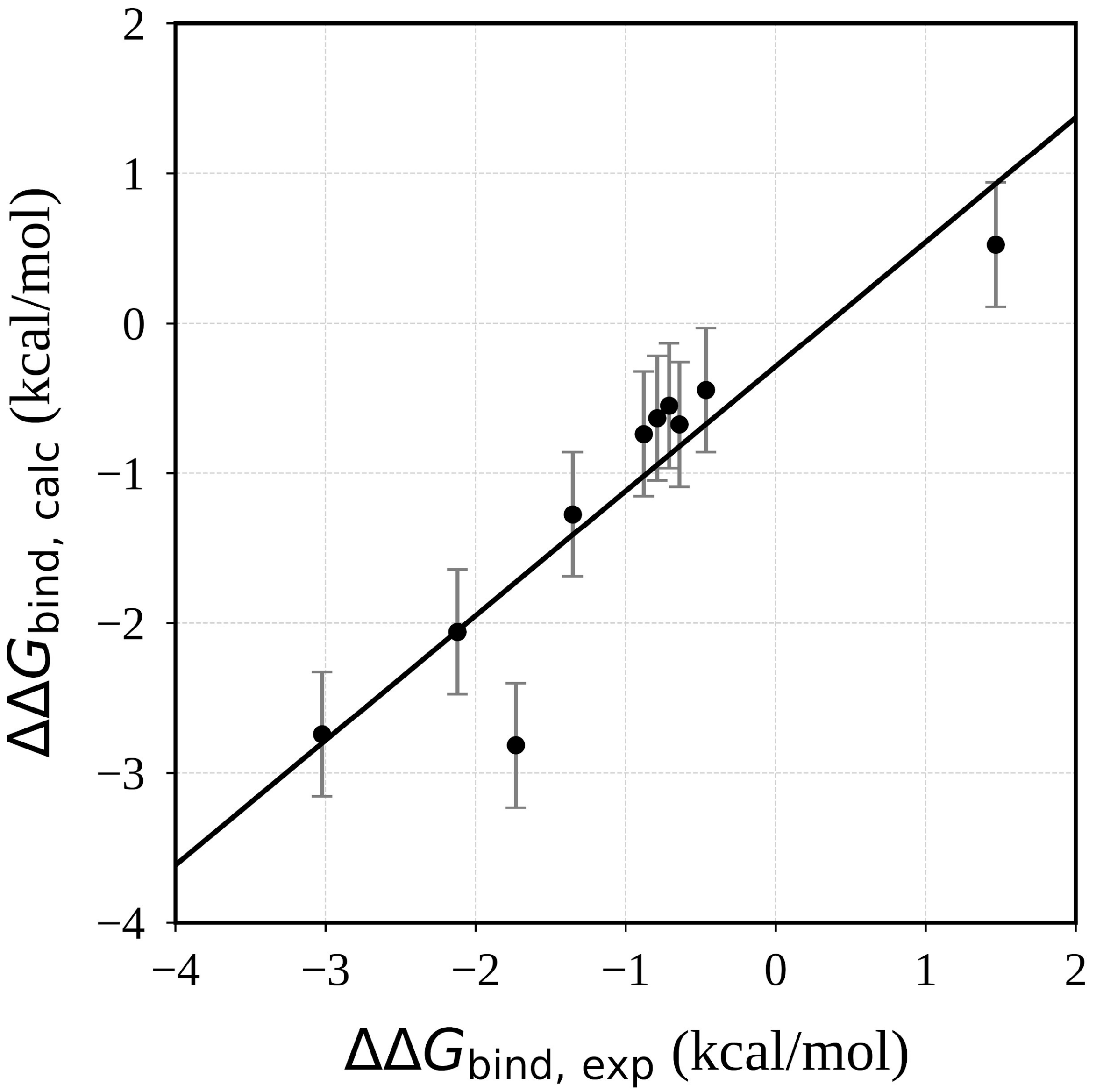

Figure S3. Plots of mean non-bonded interaction energies for TzDs, calculated from ten 2-ns molecular dynamics replicas for both the TYR–ligand complex (cplx) and the free ligand in aqueous solution (free). Electrostatic (ele) and van der Waals (vdW) energy components are reported as averages across all simulations. Electrostatic energies are shown in blue (free) and purple (cplx), while van der Waals energies are represented in green (free) and red (cplx).

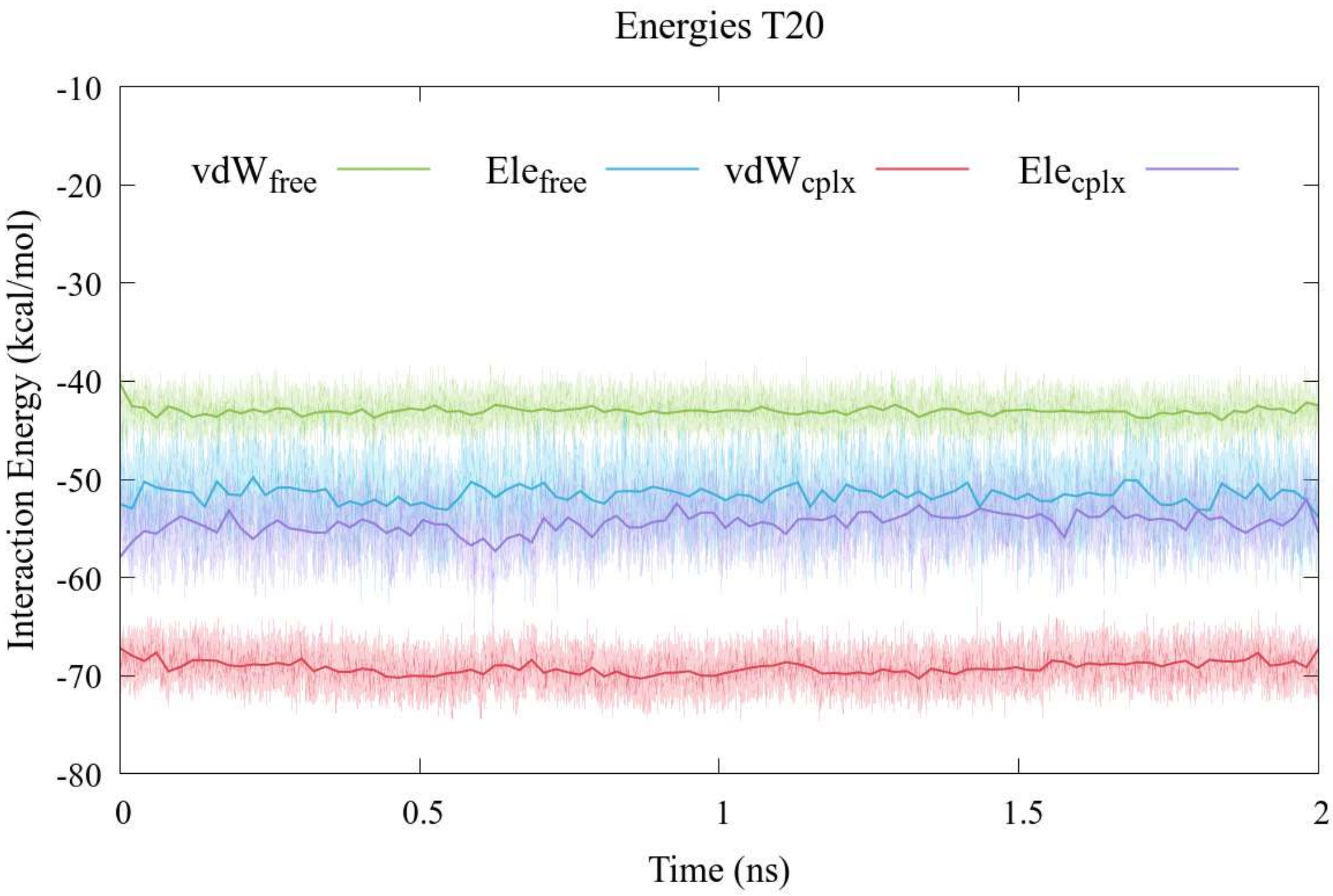

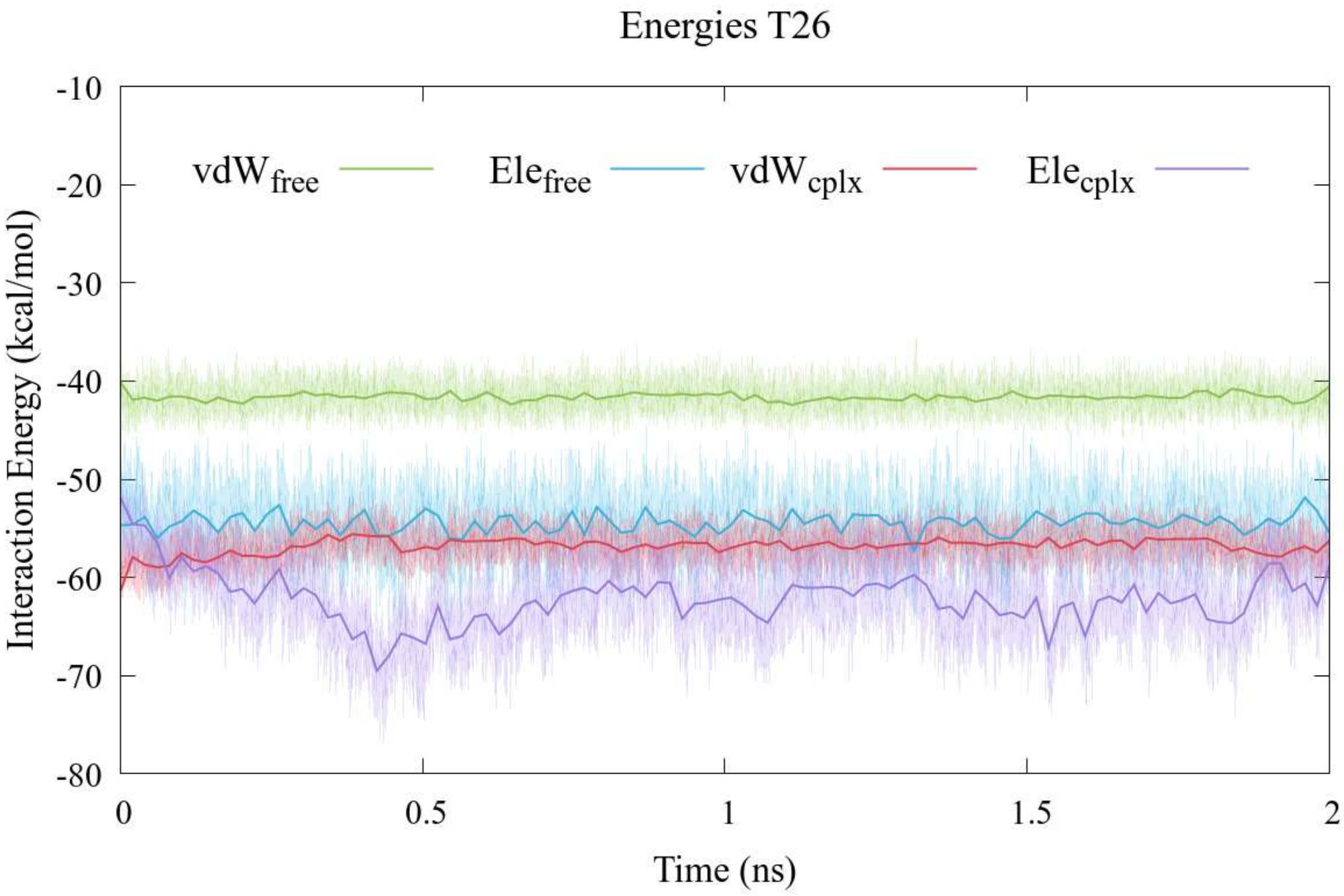

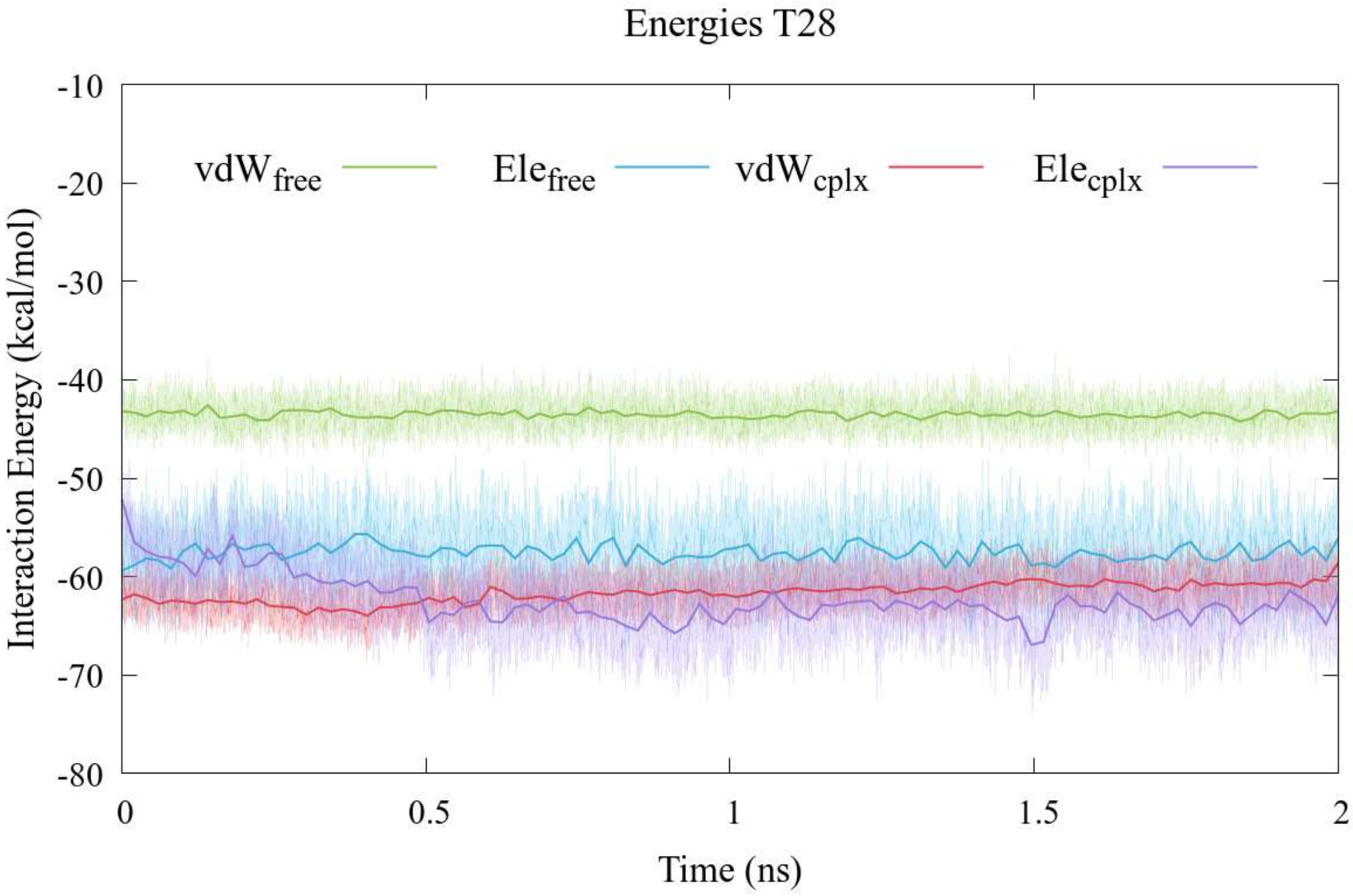

Energies T28
vdW_free
Ele_free
vdW_cplx
Ele_cplx
Interaction Energy (kcal/mol)
Time (ns)
-10
-20
-30
-40
-50
-60
-70
-80
0
0.5
1
1.5
2

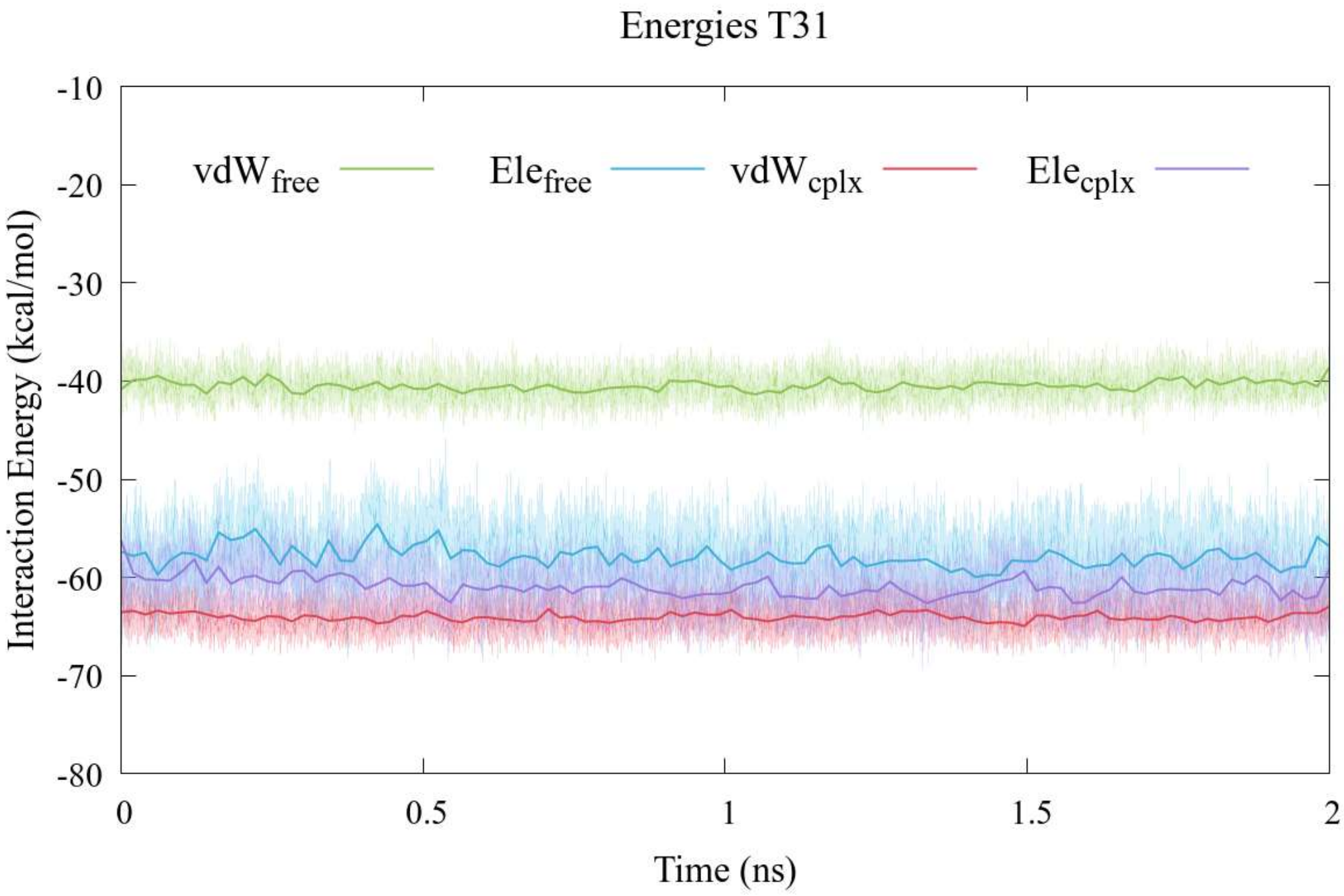

Energies T31
vdW_free
Ele_free
vdW_cplx
Ele_cplx
Interaction Energy (kcal/mol)
Time (ns)
-10
-20
-30
-40
-50
-60
-70
-80
0
0.5
1
1.5
2

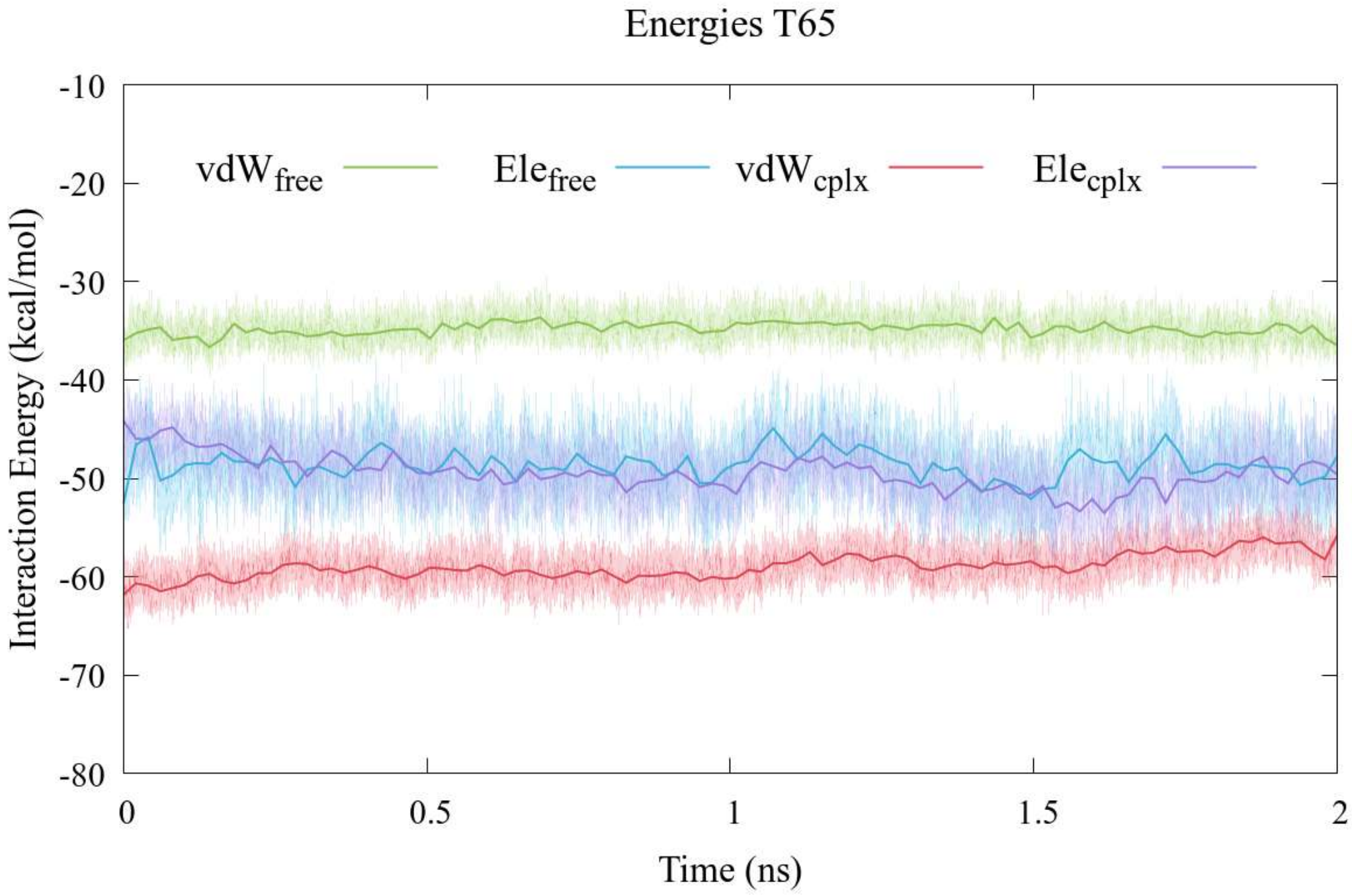

Energies T65
Interaction Energy (kcal/mol)
Time (ns)
vdW_free
Ele_free
vdW_cplx
Ele_cplx
-10
-20
-30
-40
-50
-60
-70
-80
0
0.5
1
1.5
2

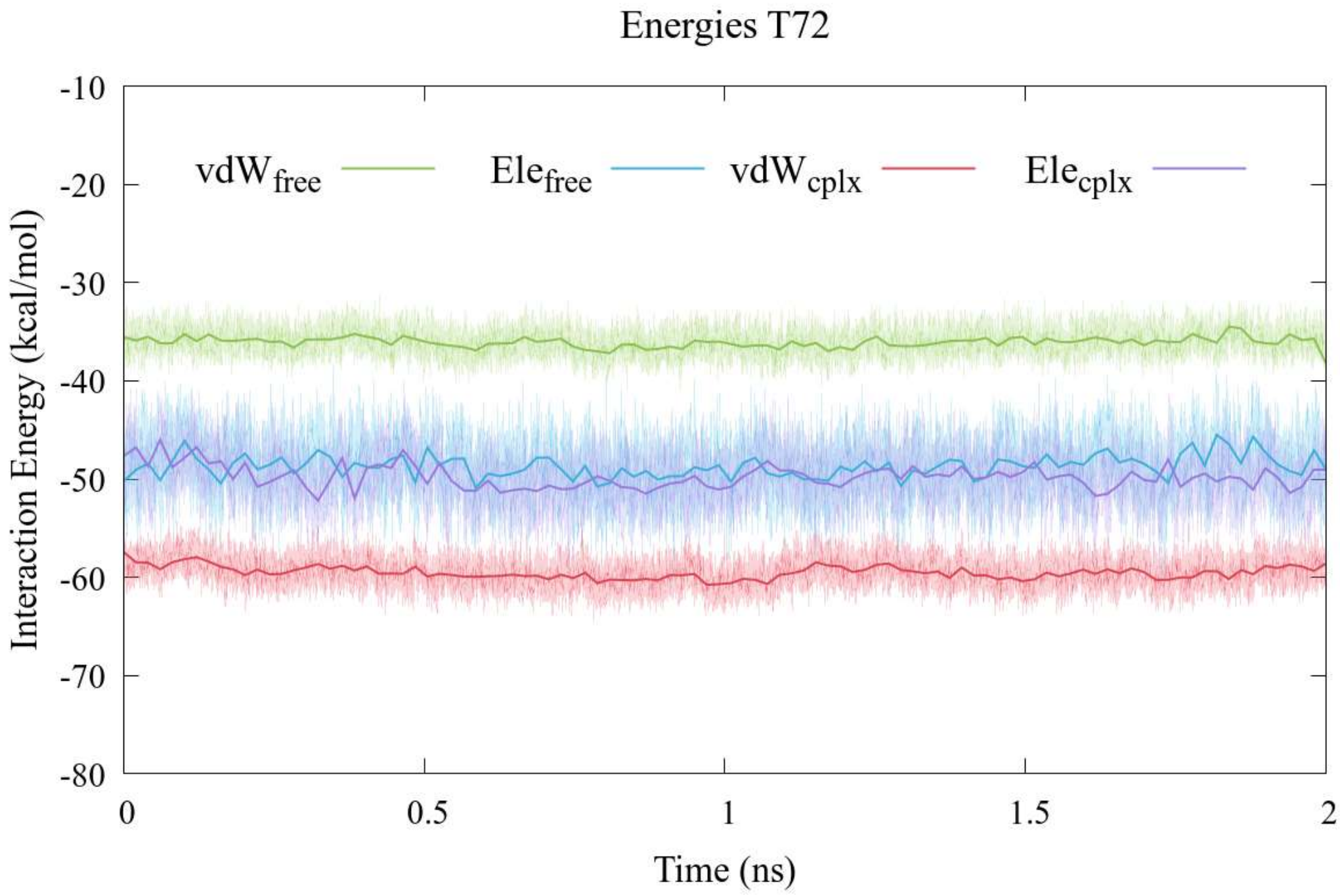

Energies T72
Interaction Energy (kcal/mol)
Time (ns)
vdW_free
Ele_free
vdW_cplx
Ele_cplx
-10
-20
-30
-40
-50
-60
-70
-80
0
0.5
1
1.5
2

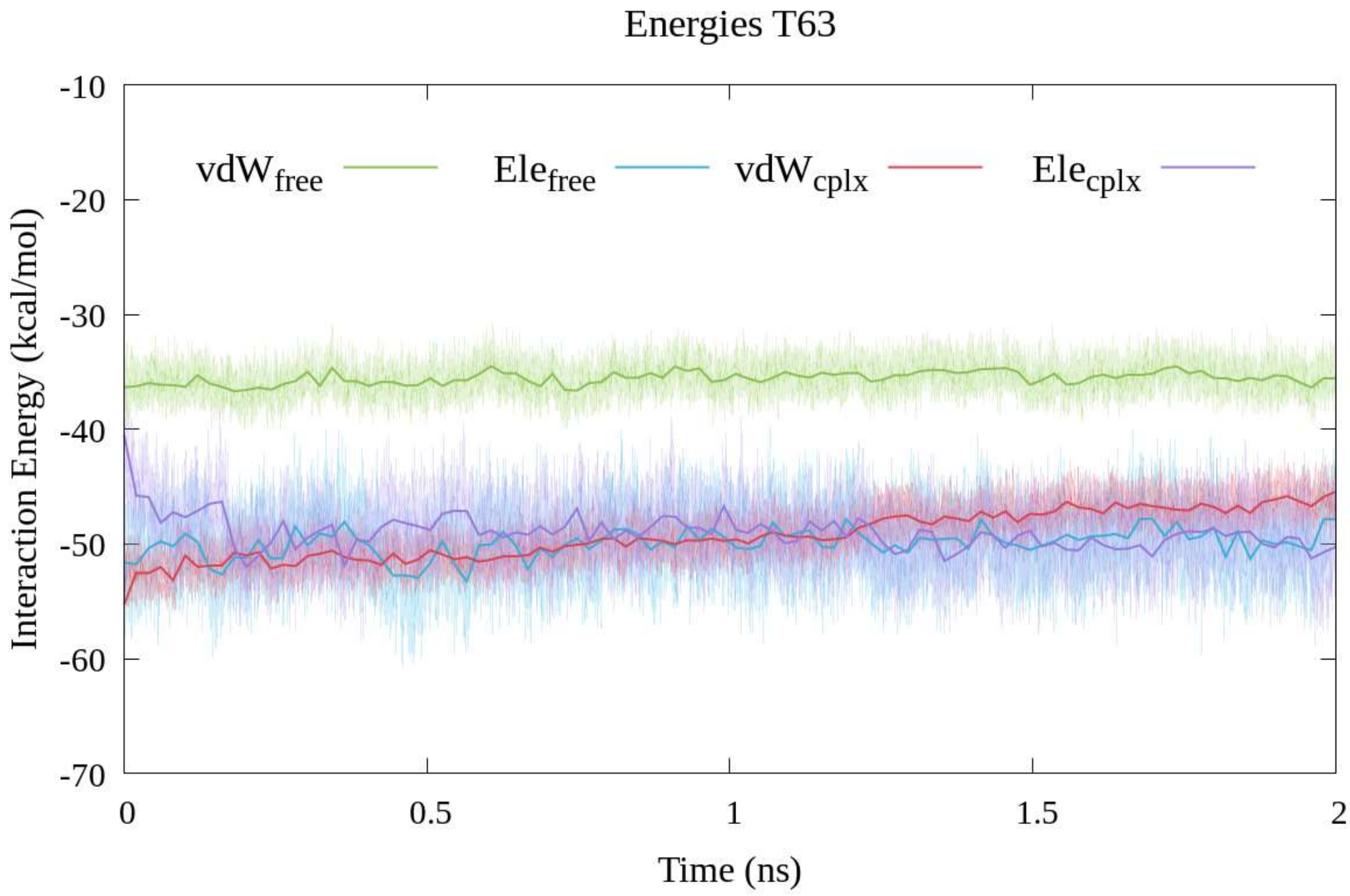
Energies T63
vdW_{free}
Ele_{free}
vdW_{cplx}
Ele_{cplx}
Interaction Energy (kcal/mol)
Time (ns)
-10
-20
-30
-40
-50
-60
-70
0
0.5
1
1.5
2

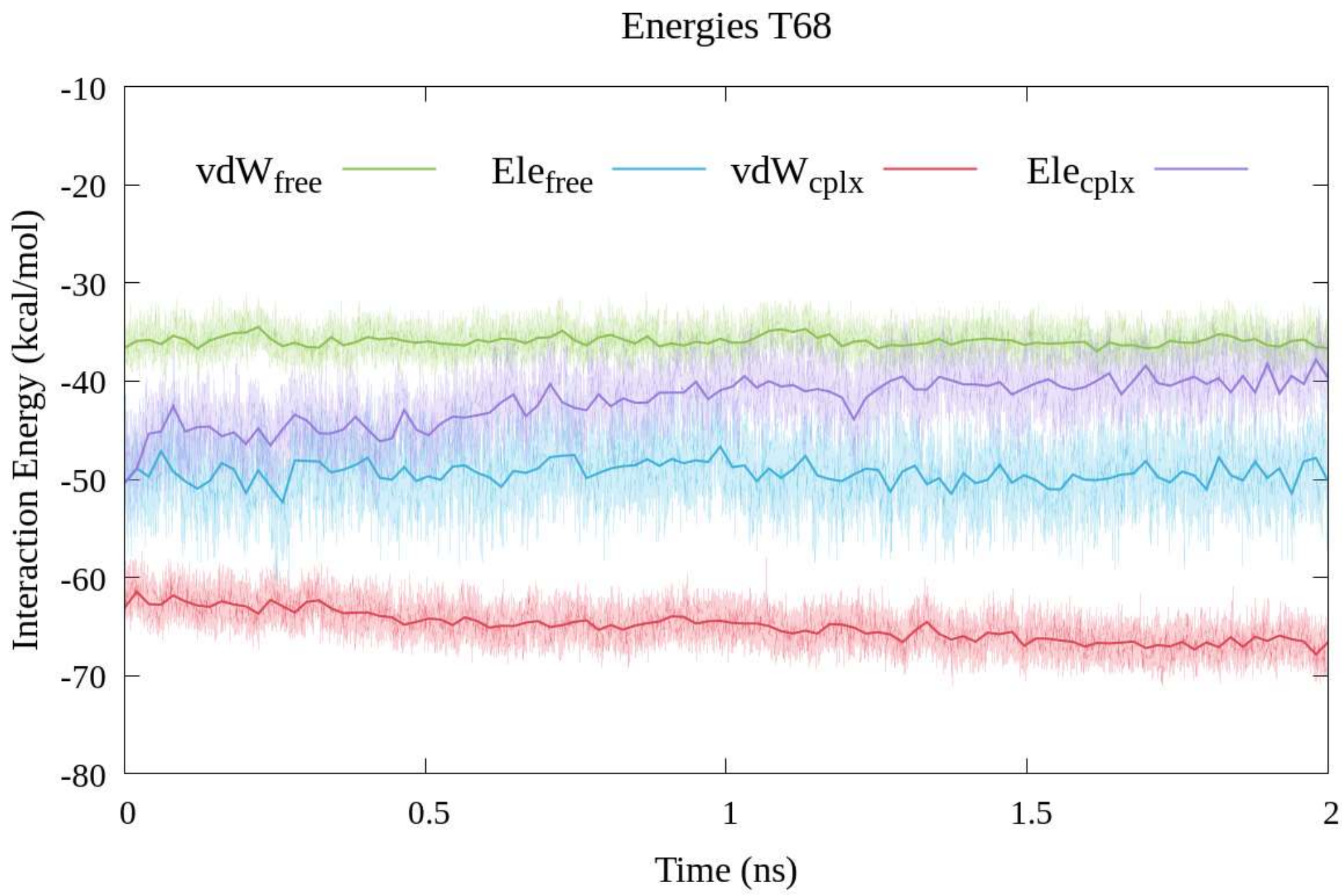
Energies T68
vdW_{free}
Ele_{free}
vdW_{cplx}
Ele_{cplx}
Interaction Energy (kcal/mol)
Time (ns)
-10
-20
-30
-40
-50
-60
-70
-80
0
0.5
1
1.5
2

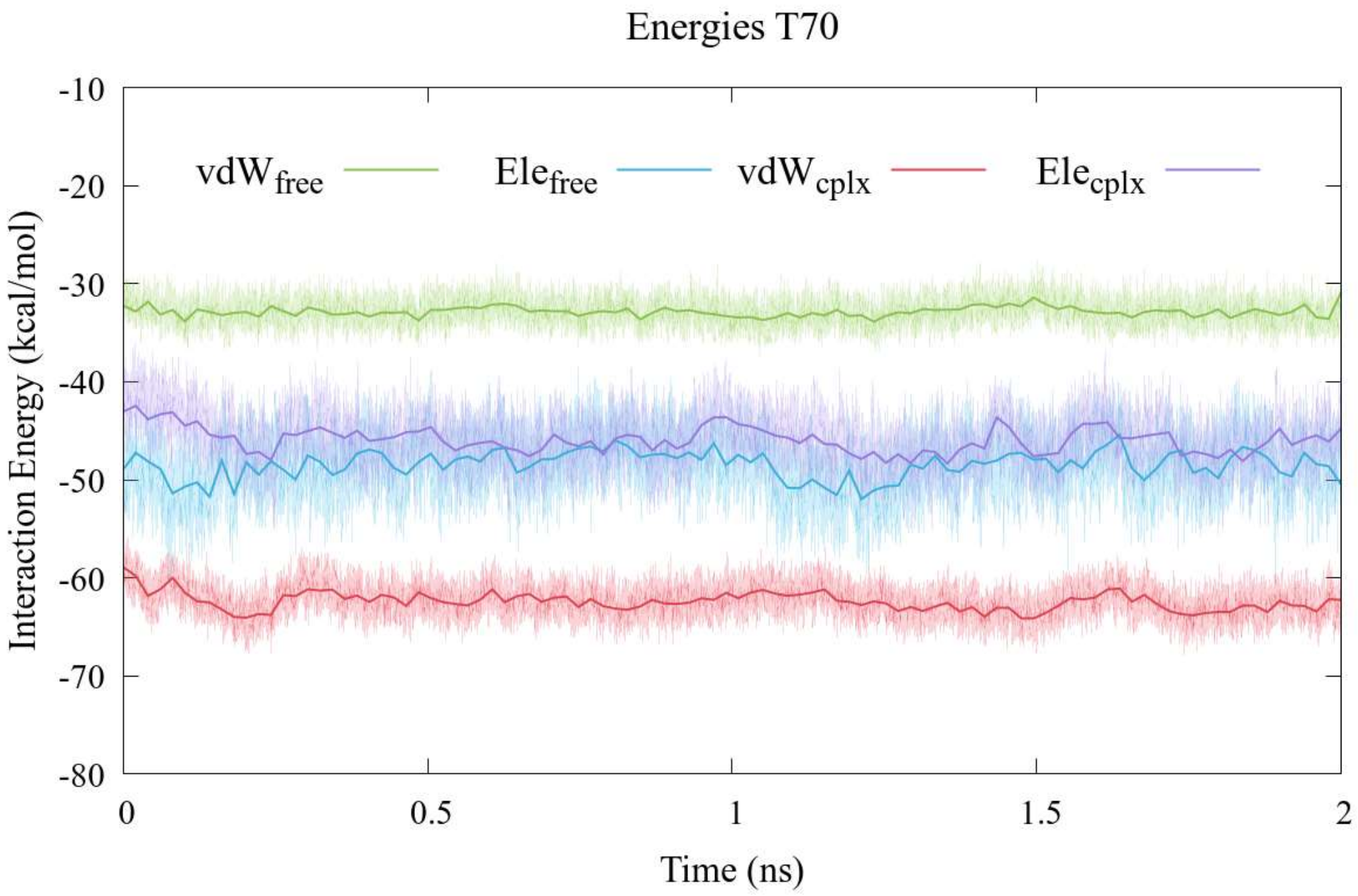
Energies T70
Interaction Energy (kcal/mol)
Time (ns)
vdWfree
Elefree
vdWcplx
Elecplx
-10
-20
-30
-40
-50
-60
-70
-80
0
0.5
1
1.5
2

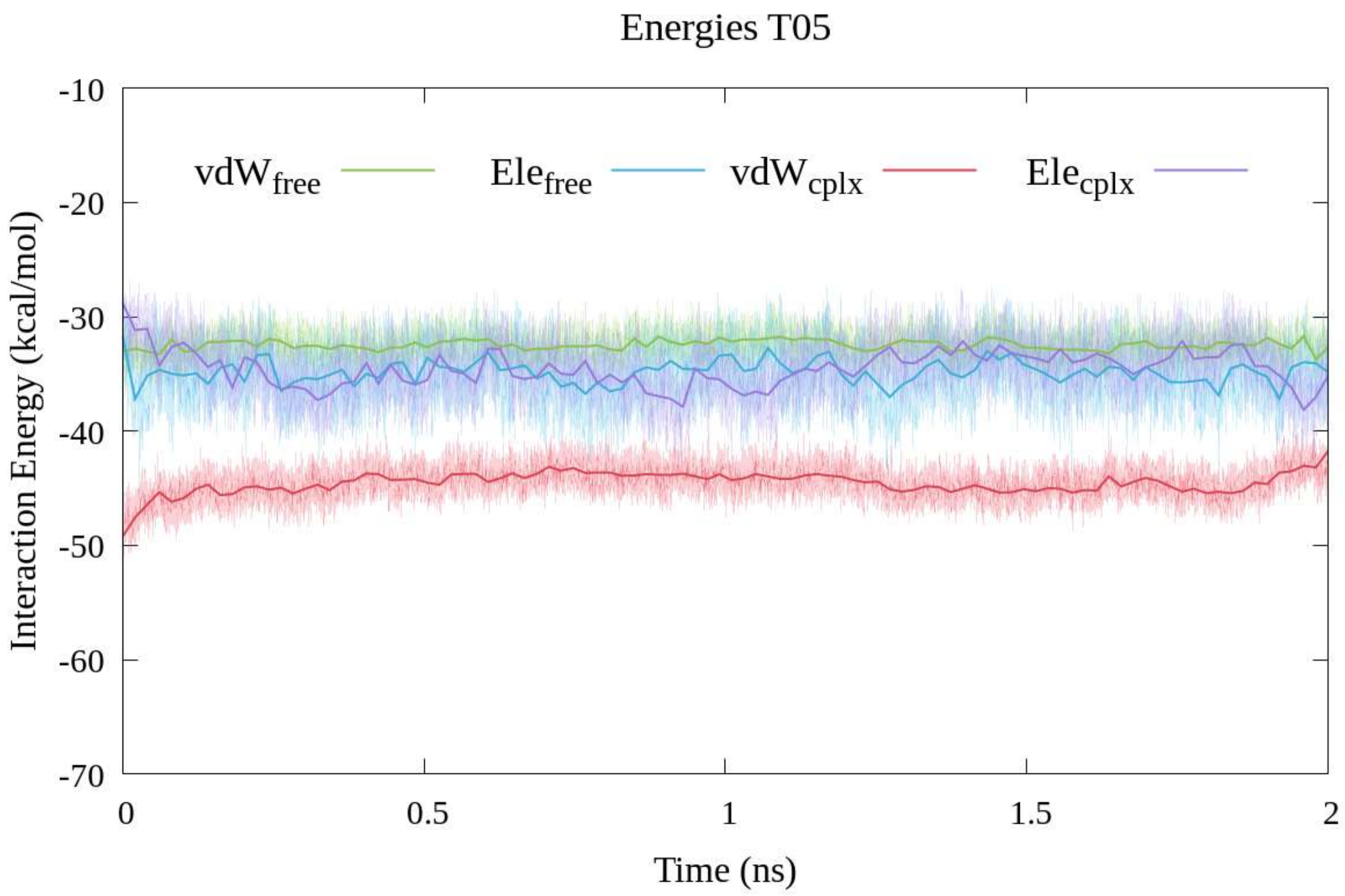
Energies T05
Interaction Energy (kcal/mol)
Time (ns)
vdWfree
Elefree
vdWcplx
Elecplx
-10
-20
-30
-40
-50
-60
-70
0
0.5
1
1.5
2

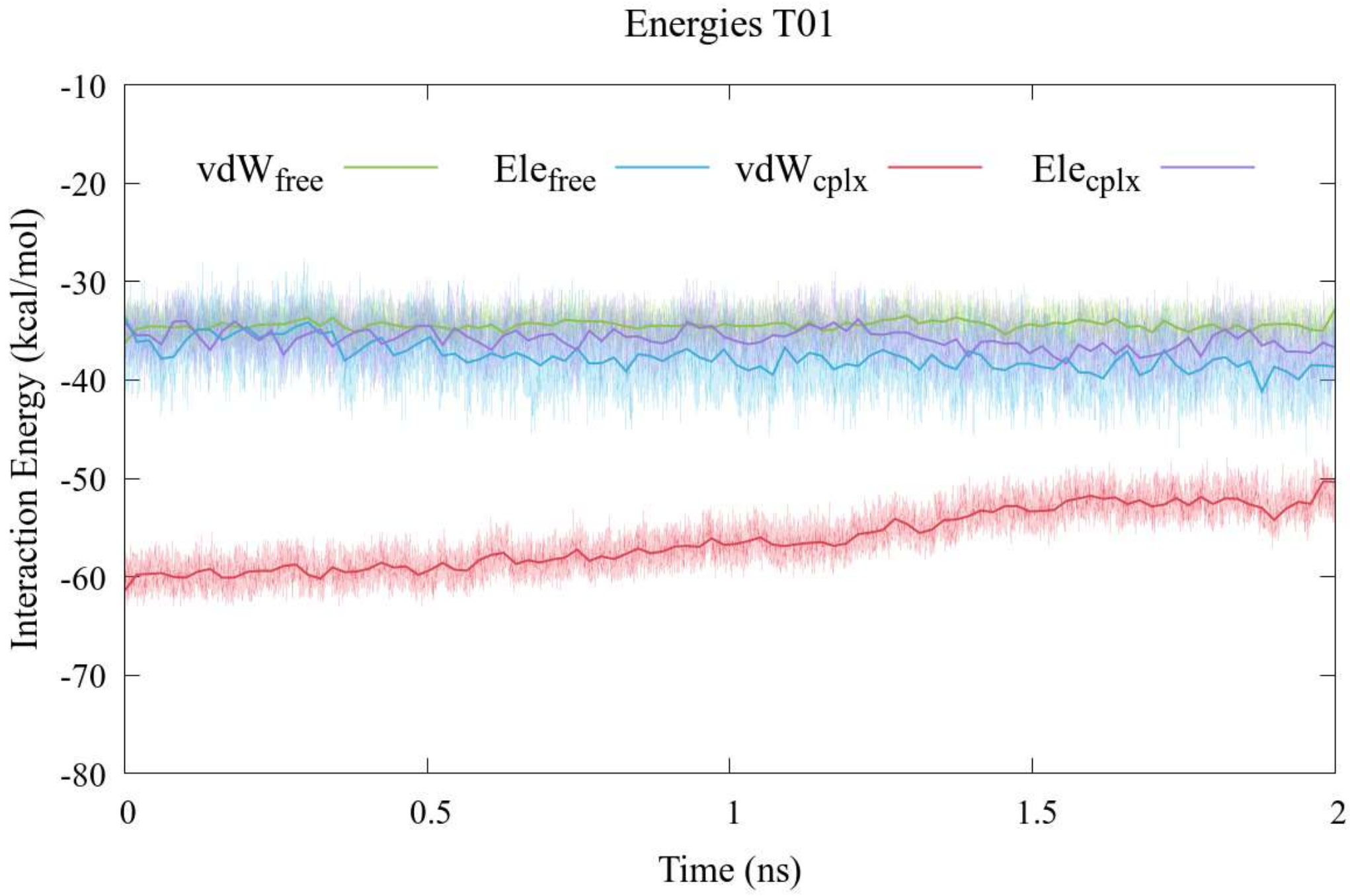
Energies T01
vdW free
Ele free
vdW cplx
Ele cplx
Interaction Energy (kcal/mol)
Time (ns)
-10
-20
-30
-40
-50
-60
-70
-80
0
0.5
1
1.5
2

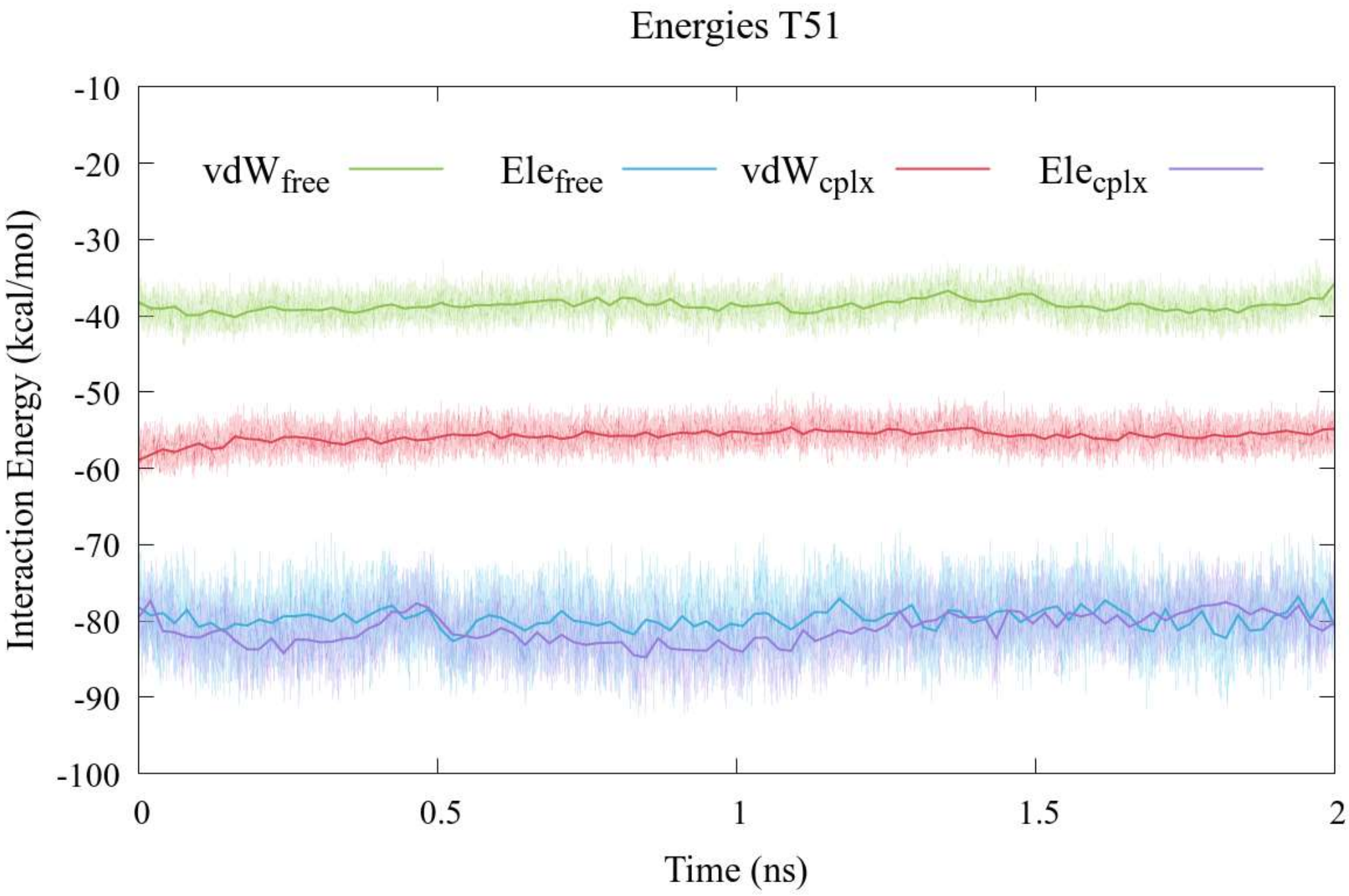
Energies T51
vdW free
Ele free
vdW cplx
Ele cplx
Interaction Energy (kcal/mol)
Time (ns)
-10
-20
-30
-40
-50
-60
-70
-80
-90
-100
0
0.5
1
1.5
2

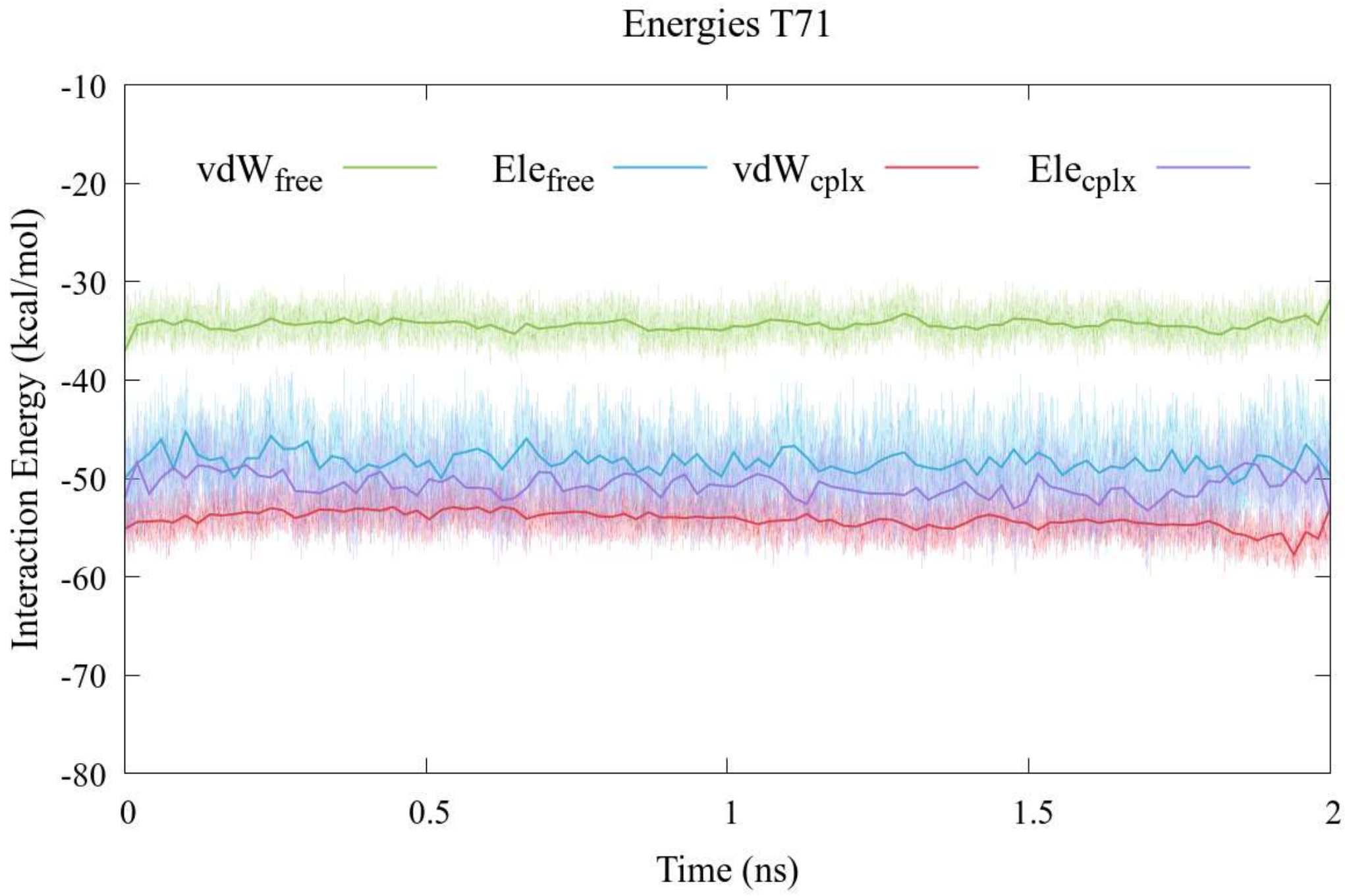
Energies T71
vdW$_{free}$
Ele$_{free}$
vdW$_{cplx}$
Ele$_{cplx}$
Interaction Energy (kcal/mol)
Time (ns)
-10
-20
-30
-40
-50
-60
-70
-80
0
0.5
1
1.5
2

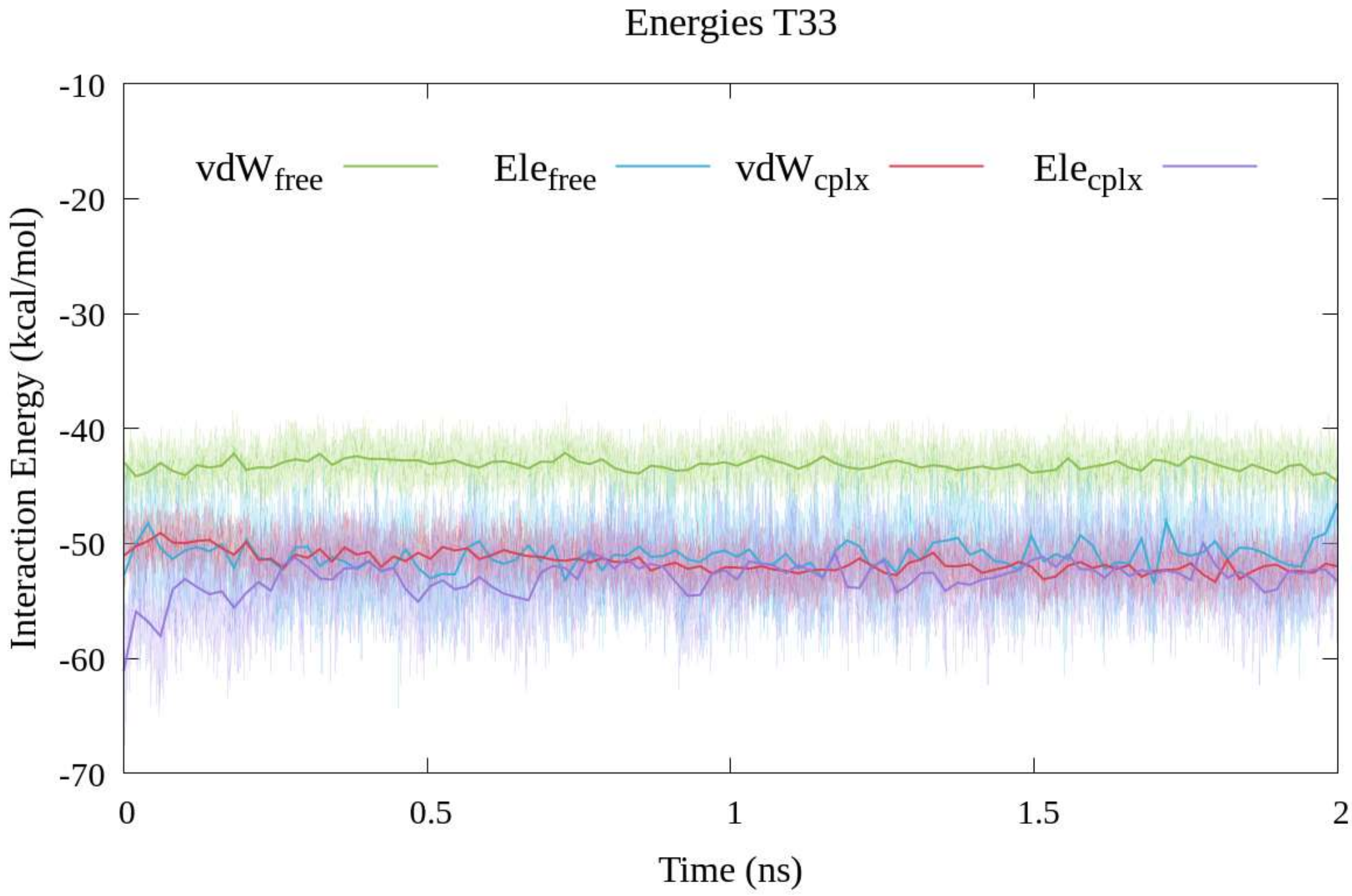
Energies T33
vdW$_{free}$
Ele$_{free}$
vdW$_{cplx}$
Ele$_{cplx}$
Interaction Energy (kcal/mol)
Time (ns)
-10
-20
-30
-40
-50
-60
-70
0
0.5
1
1.5
2

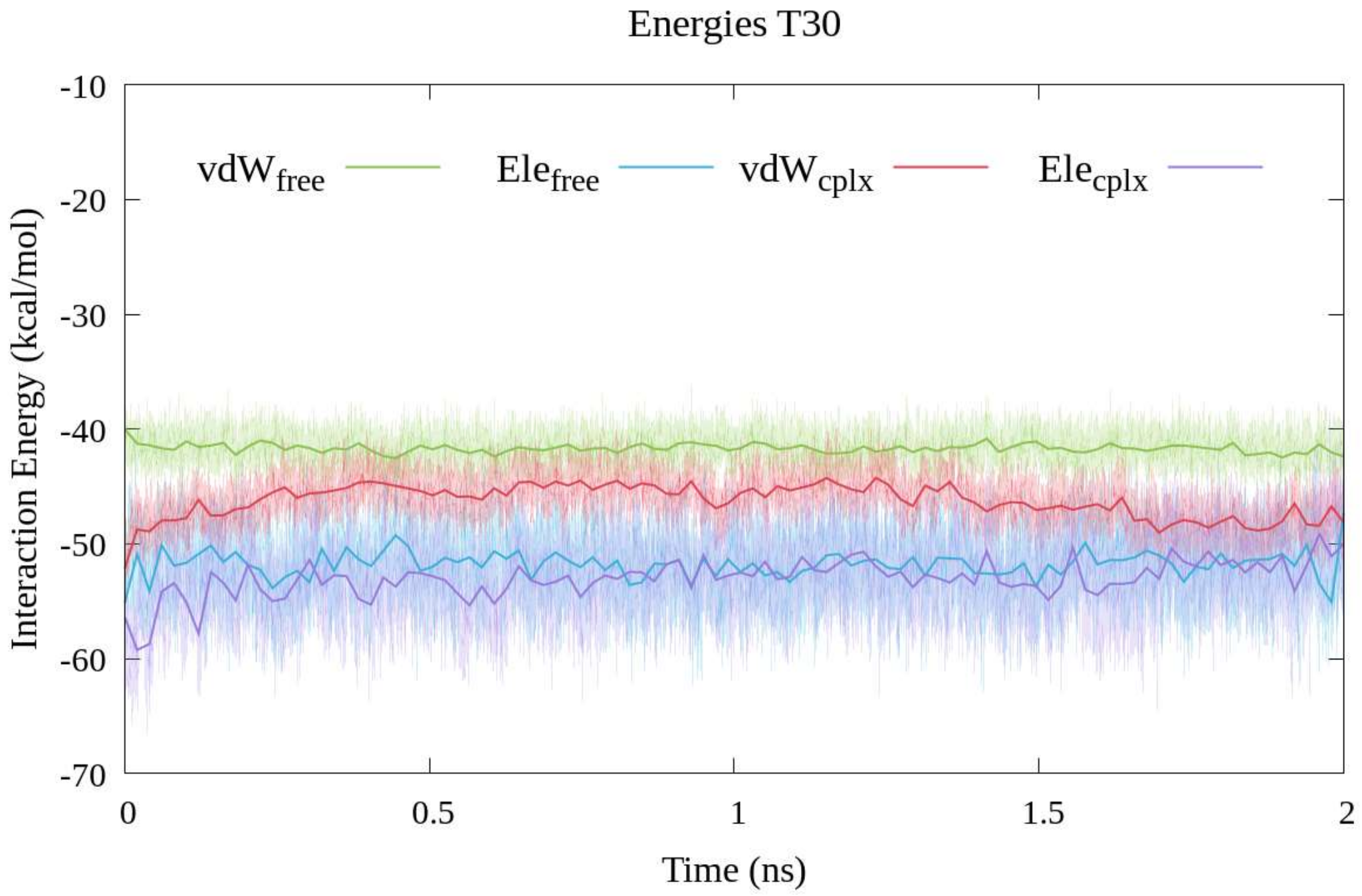

Table S2. Residue decomposition analysis of TYR–TzD complexes for inhibitors T20, T05, and T30. Energy contributions are reported in kilocalories per mole (kcal/mol) and include key per-residue interactions contributing to ligand binding.

Residue Name	Residue Number	T20		T05		T30	
		Average vdW	Average ele	Average vdW	Average ele	Average vdW	Average ele
TYR	5	0	0	0	0	0	0
ARG	6	0	-0.01	0	-0.01	0	0
VAL	7	0	0	0	0	0	0
ARG	8	0	-0.01	0	-0.02	0	0
LYS	9	0	0	0	0.01	0	0.01
ASN	10	0	-0.01	0	-0.01	0	0
VAL	11	0	-0.01	0	-0.02	0	-0.01
LEU	12	0	0	0	-0.01	0	0
HIS	13	0	0	0	-0.01	0	-0.01
LEU	14	0	0	0	0	0	0
THR	15	0	0	0	0.01	0	0.01
ASP	16	0	0	0	0	0	0
THR	17	0	0	0	0	0	0
GLU	18	0	0	0	0	0	0
LYS	19	0	0	0	0	0	0

ARG	20	0	0	0	0	0	0.01
ASP	21	0	0	0	0	0	0
PHE	22	0	0	0	0	0	0
VAL	23	0	0	0	0	0	0.01
ARG	24	0	0	0	0	0	0.01
THR	25	0	0	0	0	0	0
VAL	26	0	0	0	-0.01	0	0
LEU	27	0	0	0	0	0	0
ILE	28	0	0	0	0	0	0
LEU	29	0	0	0	-0.01	0	0
LYS	30	0	-0.01	0	-0.01	0	0
GLU	31	0	-0.01	0	-0.01	0	0
LYS	32	0	0	0	-0.01	0	0
GLY	33	0	0	0	0	0	0
ILE	34	0	0	0	0	0	0.01
TYR	35	-0.01	0.01	0	0.01	0	0
ASP	36	0	-0.01	0	0	0	0.02
ARG	37	0	-0.01	0	0	0	0.02
TYR	38	-0.02	0.01	0	0.01	0	0.02
ILE	39	-0.03	-0.01	0	0.02	-0.01	0.04
ALA	40	-0.01	-0.01	0	0.01	-0.01	0.04
TRP	41	-0.02	0	0	0	-0.01	0.03
HIS	42	-1.38	0.74	-0.02	0.08	-0.05	-0.03
GLY	43	-0.04	0.01	-0.01	0.01	-0.03	0.07
ALA	44	-0.01	-0.01	0	-0.01	-0.02	0.04
ALA	45	-0.03	0	-0.01	-0.02	-0.02	0
GLY	46	-0.07	0.03	-0.02	-0.03	-0.12	0.07
LYS	47	-0.03	0	-0.02	-0.01	-0.17	0.01
PHE	48	-0.01	0.01	-0.01	0.01	-0.04	0.01
HIS	49	-0.02	-0.06	-0.06	-0.01	-1.10	-0.09
THR	50	-0.01	0.01	-0.01	0.03	-0.02	0.02
PRO	51	0	0	-0.01	-0.02	-0.03	0.01
PRO	52	0	0.01	-0.01	0.01	-0.05	-0.03
GLY	53	0	-0.01	-0.01	-0.04	-0.26	-0.02

SER	54	-0.01	-0.01	-0.04	0.02	-0.42	0.09
ASP	55	-0.05	0.07	-1.10	0.02	-2.15	0.07
ARG	56	-0.02	-0.05	-0.07	-0.11	-0.19	0.04
ASN	57	-0.1	-0.02	-0.07	0.02	-0.18	-0.25
ALA	58	-0.02	0.02	-0.01	0.02	-0.01	-0.03
ALA	59	-0.18	0.04	-0.01	0.03	-0.02	-0.05
HIS	60	-0.53	0.32	-0.1	-0.12	-0.13	-0.1
MET	61	-0.5	-0.28	-1.78	-0.87	-1.44	0.11
SER	62	-0.02	0.01	-0.02	0.05	-0.02	0.02
SER	63	-0.01	0	0	0.01	0	0
ALA	64	-0.01	0.01	0	0	0	0
PHE	65	-0.45	0.02	-0.02	0	-0.02	0.01
LEU	66	-0.03	0.02	0	0.01	0	0.02
PRO	67	-0.01	0	0	0	0	0
TRP	68	-0.11	-0.01	-0.01	-0.01	-0.01	0
HIS	69	-0.69	0.62	-0.01	0.01	-0.01	0.06
ARG	70	-0.01	-0.01	0	-0.01	0	0
GLU	71	-0.01	-0.02	0	-0.02	0	-0.01
TYR	72	-0.07	-0.02	0	-0.01	0	0
LEU	73	-0.02	-0.02	0	-0.02	0	0
LEU	74	0	-0.01	0	-0.01	0	0
ARG	75	0	-0.02	0	-0.03	0	-0.03
PHE	76	-0.01	-0.01	0	-0.01	0	-0.01
GLU	77	0	-0.01	0	-0.01	0	0
ARG	78	0	-0.01	0	-0.01	0	0
ASP	79	0	-0.01	0	-0.01	0	0
LEU	80	0	-0.01	0	-0.01	0	-0.01
GLN	81	0	0	0	-0.01	0	-0.01
SER	82	0	0	0	0	0	0
ILE	83	0	0	0	-0.01	0	-0.01
ASN	84	0	0	0	0	0	0
PRO	85	0	0	0	0	0	0
GLU	86	0	0	0	0	0	0
VAL	87	0	0	0	0	0	0

THR	88	0	0	0	0	0	0
LEU	89	0	0	0	0	0	-0.01
PRO	90	0	0.01	0	0.01	0	0.01
TYR	91	0	-0.02	0	-0.03	0	-0.01
TRP	92	-0.02	0.01	0	0.02	0	0
GLU	93	0	0	0	-0.02	0	-0.01
TRP	94	-0.02	0	-0.01	0	0	-0.01
GLU	95	0	0	0	-0.01	0	0
THR	96	0	-0.01	0	-0.02	0	-0.01
ASP	97	0	0	0	-0.01	0	0
ALA	98	0	-0.01	0	-0.02	0	0
GLN	99	0	-0.01	0	-0.03	0	-0.01
MET	100	0	0.01	0	0.02	0	0
GLN	101	0	0.01	0	0.02	0	0
ASP	102	0	-0.01	0	-0.02	0	0
PRO	103	0	-0.01	0	0	0	0.01
SER	104	0	0	0	-0.01	0	0
GLN	105	0	-0.01	0	-0.01	0	0.01
SER	106	0	-0.01	0	-0.02	0	0
GLN	107	0	0	0	0	0	0
ILE	108	0	-0.01	0	-0.01	0	0
TRP	109	-0.01	-0.01	0	-0.02	0	0
SER	110	0	0	0	0.01	0	0.01
ALA	111	0	0	0	0	0	0
ASP	112	0	0	0	0	0	0
PHE	113	0	0	0	-0.01	0	0
MET	114	0	0	0	0.02	0	0.02
GLY	115	0	0.01	0	0.02	0	0.01
GLY	116	0	0	0	0.01	0	0.02
ASN	117	0	-0.01	0	0.01	0	0.02
GLY	118	0	0.01	0	0	0	-0.01
ASN	119	0	0	0	0	0	0
PRO	120	0	-0.01	0	-0.01	0	0
ILE	121	0	0	0	-0.01	0	0

LYS	122	0	0	0	0	0	0.01
ASP	123	-0.01	-0.01	0	0.01	0	0.04
PHE	124	-0.01	0	0	0.02	-0.01	0.02
ILE	125	0	0.01	0	0	0	-0.01
VAL	126	0	-0.01	0	-0.01	0	0
ASP	127	0	0	0	-0.02	0	-0.01
THR	128	0	0	0	0.01	0	0.02
GLY	129	0	-0.01	0	-0.01	0	0
PRO	130	0	0	0	-0.01	0	0
PHE	131	0	0	0	0	0	0.01
ALA	132	0	0	0	0	0	0.01
ALA	133	0	0.01	0	0	0	-0.01
GLY	134	0	0	0	0	0	-0.01
ARG	135	0	0	0	0	0	0
TRP	136	0	0	0	-0.01	0	-0.01
THR	137	0	0	0	0.02	0	0.01
THR	138	-0.01	0.01	0	-0.03	0	-0.04
ILE	139	-0.01	-0.01	0	0.02	-0.01	0.03
ASP	140	-0.01	0.03	0	0.02	-0.05	-0.06
GLU	141	-0.05	0.05	-0.02	0.03	-0.89	0
GLN	142	-0.01	0.05	0	0.02	-0.04	0
GLY	143	0	0.01	0	0	0	-0.02
ASN	144	0	0	0	0.02	-0.01	0.03
PRO	145	0	-0.01	0	-0.01	0	0
SER	146	0	0	0	0	-0.01	0.01
GLY	147	0	0	0	0	0	0
GLY	148	0	-0.01	0	-0.01	0	0.02
LEU	149	-0.01	0	0	-0.02	-0.01	-0.03
LYS	150	-0.02	-0.01	-0.01	0.02	-0.02	0.02
ARG	151	-0.02	-0.02	-0.01	-0.05	-0.01	-0.01
ASN	152	-0.02	0.03	-0.01	0.04	-0.01	-0.01
PHE	153	-0.01	-0.01	-0.01	-0.05	0	-0.01
GLY	154	-0.01	-0.03	-0.01	-0.08	0	-0.01
ALA	155	-0.02	-0.01	-0.02	-0.06	-0.01	-0.02

THR	156	-0.17	0.01	-0.13	0.12	-0.04	0.02
LYS	157	-0.07	-0.02	-0.05	0.05	-0.01	0.02
GLU	158	-2.49	0.1	-2.07	-0.83	-0.18	-0.14
ALA	159	-0.1	0	-0.09	0	-0.02	0.01
PRO	160	-0.02	-0.01	-0.02	0	-0.01	0.02
THR	161	-0.01	0.04	-0.01	0.08	0	0
LEU	162	-0.01	0	-0.01	-0.04	0	-0.02
PRO	163	-0.01	-0.02	-0.01	-0.03	0	0.01
THR	164	0	0.01	0	0.04	0	0.01
ARG	165	0	0	0	0.01	0	0
ASP	166	0	0.01	0	0.01	0	0
ASP	167	-0.01	0	0	0	0	0
VAL	168	0	0	0	0	0	0
LEU	169	0	0	0	0	0	0
ASN	170	0	-0.02	0	-0.04	0	0.01
ALA	171	0	-0.01	0	-0.02	0	0
LEU	172	0	0	0	0	0	0
LYS	173	0	-0.01	0	-0.02	0	0
ILE	174	0	0	0	0	0	0.01
THR	175	0	0	0	0	0	0
GLN	176	0	0.01	0	0.02	0	0
TYR	177	0	0.02	0	0.06	0	0
ASP	178	0	-0.01	0	0	0	0.02
THR	179	0	0.01	0	0.01	0	-0.01
PRO	180	0	0.01	0	0.01	0	-0.01
PRO	181	0	0	-0.01	0	-0.01	0.01
TRP	182	-0.01	0	-0.01	-0.02	-0.01	0.02
ASP	183	-0.02	-0.01	-0.05	-0.01	-0.04	-0.01
MET	184	-0.16	0.04	-1.43	-0.26	-0.66	-0.21
THR	185	-0.02	0.01	-0.06	0.06	-0.04	0.03
SER	186	-0.01	0.02	-0.01	0.04	-0.01	-0.01
GLN	187	-0.01	-0.01	-0.01	0.01	-0.01	0.03
ASN	188	0	-0.01	0	-0.01	0	0.01
SER	189	0	0.01	0	0.02	0	0

PHE	190	-0.01	0.02	0	0.06	0	0
ARG	191	-0.03	0.04	-0.02	0.18	-0.01	0.04
ASN	192	-0.03	0.08	-0.04	0.27	-0.02	0.03
GLN	193	-0.02	0.06	-0.01	0.18	-0.01	0
LEU	194	-0.03	0.02	-0.01	0.07	-0.01	0.01
GLU	195	-0.25	0	-0.09	0.18	-0.04	0.14
GLY	196	-0.1	0.12	-0.11	0.3	-0.02	-0.02
PHE	197	-0.58	-0.01	-1.69	-0.2	-0.29	-0.03
ILE	198	-0.04	0.03	-0.05	0.07	-0.01	0
ASN	199	-0.13	0	-0.08	0	-0.01	0.01
GLY	200	-0.82	-0.14	-0.51	-0.5	-0.04	-0.03
PRO	201	-1.51	-0.12	-1.80	-0.33	-0.11	0
GLN	202	-0.1	0.06	-0.1	0.27	-0.02	0.05
LEU	203	-0.04	0.03	-0.02	0.12	-0.01	0
HIS	204	-1.60	0.37	-0.11	-0.25	-0.05	0.09
ASN	205	-1.30	-0.47	-0.8	-0.48	-0.25	0.29
ARG	206	-0.17	-0.02	-0.13	0.02	-0.04	0.03
VAL	207	-0.13	0	-0.03	-0.04	-0.02	-0.01
HIS	208	-1.42	-0.13	-0.27	-0.4	-0.14	-0.09
ARG	209	-5.90	-1.89	-3.93	-2.20	-0.95	-0.04
TRP	210	-0.07	0.02	-0.04	-0.04	-0.02	0
VAL	211	-0.07	0	-0.02	-0.09	-0.01	-0.03
GLY	212	-0.08	-0.04	-0.03	-0.04	-0.03	0.01
GLY	213	-0.08	0	-0.02	0.07	-0.06	0.1
GLN	214	-0.35	0.03	-0.03	0.08	-0.09	0.11
MET	215	-0.88	-0.07	-0.07	0.12	-0.08	0.13
GLY	216	-1.25	-3.50	-0.57	0.36	-0.5	0.09
VAL	217	-2.49	0.55	-0.8	0.19	-2.75	-0.74
VAL	218	-4.18	-0.07	-0.35	0.02	-1.14	-0.78
PRO	219	-0.42	0.05	-0.06	0.02	-1.07	-0.09
THR	220	-0.2	0.02	-0.02	0.03	-0.23	-0.04
ALA	221	-0.22	0.07	-0.02	-0.05	-0.04	-0.11
PRO	222	-0.26	0.03	-0.01	-0.01	-0.02	-0.04
ASN	223	-0.03	0.05	0	-0.04	-0.01	-0.11

ASP	224	-0.04	0	0	0.03	-0.01	0.02
PRO	225	-0.01	0.01	0	0.01	0	0
VAL	226	-0.03	0.02	0	0.01	0	-0.01
PHE	227	-1.33	0.2	-0.01	0.04	-0.01	-0.02
PHE	228	-0.04	0.03	0	0.03	0	0.01
LEU	229	-0.01	0.02	0	0	0	-0.01
HIS	230	-0.33	0.06	-0.01	0.04	-0.01	0.02
HIS	231	-0.6	0.6	-0.01	0.09	-0.01	0.05
ALA	232	-0.01	0.01	0	0	0	-0.01
ASN	233	-0.01	-0.02	0	-0.07	0	-0.02
VAL	234	-0.04	-0.02	0	-0.03	0	-0.02
ASP	235	-0.01	-0.01	0	-0.02	0	-0.01
ARG	236	0	-0.02	0	-0.05	0	-0.02
ILE	237	-0.01	-0.01	0	-0.03	0	-0.01
TRP	238	-0.01	0	0	0	0	-0.01
ALA	239	0	0	0	-0.01	0	-0.01
VAL	240	0	-0.01	0	-0.02	0	0
TRP	241	0	-0.01	0	-0.02	0	0
GLN	242	0	0	0	0	0	0
ILE	243	0	0	0	-0.01	0	0
ILE	244	0	-0.01	0	-0.01	0	0
HIS	245	0	0.01	0	0.02	0	0
ARG	246	0	0	0	0	0	0
ASN	247	0	0	0	0	0	0
GLN	248	0	0	0	0	0	0
ASN	249	0	0.01	0	0.02	0	0
TYR	250	0	0	0	-0.01	0	-0.01
GLN	251	0	0	0	0	0	0
PRO	252	0	0	0	0.01	0	0.01
MET	253	0	0	0	0.01	0	0.01
LYS	254	0	0	0	0.01	0	0
ASN	255	0	0	0	0	0	-0.01
GLY	256	0	-0.01	0	-0.01	0	0
PRO	257	0	0.01	0	0.02	0	0

PHE	258	0	0	0	0	0	0.02
GLY	259	0	0.01	0	0.01	0	0.01
GLN	260	-0.01	0.02	0	0.04	-0.01	-0.02
ASN	261	0	0.01	0	0.02	0	0.01
PHE	262	0	0	0	0.01	0	0.01
ARG	263	0	0	0	0	0	0
ASP	264	0	0	0	0	0	0
PRO	265	0	0	0	0	0	0
MET	266	-0.01	0.01	0	0.01	0	0
TYR	267	-0.01	0.02	0	0.02	-0.01	0
PRO	268	-0.01	-0.01	0	-0.01	0	-0.01
TRP	269	-0.01	-0.01	0	-0.01	0	0
ASN	270	0	0	0	0	0	0.01
THR	271	0	0	0	0	0	0
THR	272	0	0.01	0	0.01	0	0
PRO	273	0	0	0	0	0	0
GLU	274	0	0	0	0	0	0
ASP	275	0	0	0	0	0	0
VAL	276	0	0	0	0.01	0	0
MET	277	0	0.01	0	0.02	0	0
ASN	278	0	0	0	0.01	0	0
HIS	279	0	-0.01	0	-0.01	0	0
ARG	280	0	0	0	0	0	0
LYS	281	0	0	0	0	0	0
LEU	282	0	0	0	-0.01	0	0
GLY	283	0	0	0	0	0	0
TYR	284	0	0	0	-0.01	0	0
VAL	285	0	0	0	0	0	0
TYR	286	0	0	0	0	0	0
ASP	287	0	0	0	-0.01	0	0
ILE	288	0	0	0	-0.01	0	0
GLU	289	0	0	0	-0.01	0	0
LEU	290	0	0	0	0	0	0
CU6A	291	-5.95	-5.54	-0.01	-2.23	-0.01	-1.96

CU6B	292	-4.98	-2.94	-0.01	-3.00	-0.01	-1.71

Table S3. Residue decomposition analysis of 15 TYR–TzD complexes. All values are reported in kilocalories per mole (kcal/mol).

Inhib.	Interac. energy components	His42	His49	Asp55	Met61	Glu158	Arg209	Gly216	Val218
T20	vdW	-1.38	-0.02	-0.50	-0.50	-2.49	-5.90	-1.25	-4.18
	Ele	0.74	-0.06	0.07	-0.28	0.10	-1.89	-3.50	-0.07
T26	vdW	-0.47	-0.03	-0.09	-0.44	-1.89	-4.11	-1.43	-2.41
	Ele	0.28	-0.07	0.06	-0.14	-0.14	-2.94	-0.75	0.41
T28	vdW	-0.54	-0.04	-0.14	-1.62	-0.53	-3.93	-0.28	-4.15
	Ele	0.50	-0.13	0.24	-0.58	-0.05	-1.40	-4.86	-0.22
T31	vdW	-0.75	-0.03	-0.20	-1.48	-2.69	-4.34	-1.39	-3.64
	Ele	1.21	-0.05	0.01	-0.71	-0.61	-3.82	-3.97	-0.52
T65	vdW	-0.60	-0.07	-0.17	-1.69	-0.74	-3.98	-1.43	-4.39
	Ele	0.15	-0.16	0.24	-1.29	-0.13	-0.23	-0.27	-0.26
T72	vdW	-0.52	-0.02	-0.15	-0.73	-1.98	-4.38	-1.23	-2.84
	Ele	0.11	-0.09	0.16	-0.56	0.06	-0.66	-0.50	-0.16
T63	vdW	-0.04	-0.01	-0.03	-0.22	-2.73	-5.44	-0.97	-1.11
	Ele	-0.01	-0.02	0.05	-0.07	-0.45	-1.92	0.09	-0.08
T68	vdW	-0.85	-0.14	-1.26	-2.41	-0.22	-2.73	-0.28	-5.62
	Ele	-0.10	-0.06	-0.55	-0.87	0.33	-0.23	-3.13	-0.69
T70	vdW	-0.59	-0.02	-0.13	-1.07	-2.35	-3.28	-1.31	-2.42
	Ele	0.01	-0.01	-0.01	-0.42	-0.53	-1.94	-0.93	-0.13
T05	vdW	-0.02	-0.06	-1.10	-1.78	-2.07	-3.93	-0.57	-0.35
	Ele	0.08	-0.01	0.02	-0.87	-0.83	-2.20	0.36	0.02
T01	vdW	-0.36	-0.02	-0.10	-0.75	-1.72	-3.08	-0.84	-2.06
	Ele	0.11	-0.05	0.24	-0.48	-0.54	-0.94	-0.23	-0.02
T51	vdW	-0.35	-0.22	-1.53	-1.58	-0.64	-3.14	-1.33	-2.60
	Ele	0.21	0.01	0.02	0.08	-0.07	-0.03	-0.39	-0.31
T71	vdW	-0.44	-0.13	-1.14	-2.05	-0.28	-3.25	-0.81	-4.54
	Ele	-0.02	-0.03	-0.55	-0.66	0.36	0.07	-2.71	-0.66
T33	vdW	-0.06	-0.11	-0.53	-0.85	-1.30	-4.27	-1.16	-1.50
	Ele	0.05	-0.04	0.06	-0.44	-0.24	-0.27	0.25	0.23
T30	vdW	-0.05	-1.10	-2.15	-1.44	-0.18	-0.95	-0.50	-1.14
	Ele	-0.03	-0.09	0.07	0.11	-0.14	-0.04	0.09	-0.78
